# Dialdehyde Alginate as a Crosslinker for Chitosan/Starch Films: Toward Biocompatible and Antioxidant Wound Dressing Materials

**DOI:** 10.3390/ijms27031174

**Published:** 2026-01-23

**Authors:** Sylwia Grabska-Zielińska, Marek Pietrzak, Lidia Zasada, Krzysztof Łukowicz, Agnieszka Basta-Kaim, Marta Michalska-Sionkowska, Marcin Wekwejt, Beata Kaczmarek-Szczepańska

**Affiliations:** 1 Faculty of Chemical Technology and Engineering, Bydgoszcz University of Science and Technology, Seminaryjna 3, 85-326 Bydgoszcz, Poland; marek.pietrzak@pbs.edu.pl; 2 Laboratory for Functional Polymeric Materials, Faculty of Chemistry, Nicolaus Copernicus University in Toruń, Gagarin 7, 87-100 Toruń, Poland; 503555@doktorant.umk.pl; 3 Laboratory of Immunoendocrinology, Department of Experimental Neuroendocrinology, Maj Institute of Pharmacology, Polish Academy of Sciences, 12 Smętna St., 31-343 Kraków, Poland; lukowicz@if-pan.krakow.pl (K.Ł.); basta@if-pan.krakow.pl (A.B.-K.); 4 Department of Medical Physics, Cyclotron Centre Bronowice, Institute of Nuclear Physics, Polish Academy of Sciences, Radzikowskiego 152, 31-342 Kraków, Poland; 5 Department of Environmental Microbiology and Biotechnology, Faculty of Biological and Veterinary Sciences, Nicolaus Copernicus University in Toruń, Lwowska 1, 87-100 Toruń, Poland; mms@umk.pl; 6 Department of Biomaterials Technology, Faculty of Mechanical Engineering and Ship Technology, Gdańsk University of Technology, 80-222 Gdańsk, Poland; marcin.wekwejt@pg.edu.pl

**Keywords:** dialdehyde alginate, starch, chitosan, wound dressing

## Abstract

Biopolymer-based films have attracted increasing attention as sustainable and bioactive materials for wound management. Among them, chitosan (CTS) and starch (ST) blend represent promising candidate due to their natural origin, biodegradability, and intrinsic biological activity; however, their mechanical weakness and limited stability necessitate additional modification. This study reports the development and characterization of CTS-ST thin films crosslinked with dialdehyde alginate (ADA), synthesized via controlled oxidation. Two ADA variants differing in aldehyde group content were prepared to investigate the effect of crosslinking on the structural, physicochemical, and biological performance of the materials. The films were fabricated by blending 2% *w*/*v* CTS and ST in varying mass ratios (75/25, 50/50, and 25/75), followed by the addition of ADA (5% *w*/*w*) and glycerol (5% *w*/*w*) as a plasticizer. The mixtures were then cast onto plates and dried under ambient conditions. Comprehensive characterization included Fourier-transform infrared spectroscopy, moisture content analysis, contact angle measurements, antioxidant activity assay, hemolysis testing, and cytotoxicity evaluation using human keratinocyte cells. The results demonstrated that both the ADA variant and CTS/ST ratio significantly influenced crosslinking efficiency, hydrophilicity, and antioxidant behavior. All samples exhibited non-hemolytic behavior and no significant cytotoxic effects, indicating their favorable biocompatibility. The combination of biostability, antioxidant ability, and absence of cytotoxic effects highlights the potential of ADA-crosslinking CTS/ST films for further development as wound dressing materials and other biomedical applications.

## 1. Introduction

Interest in wound dressings based on natural polymers has increasingly grown in recent years. Chitosan (CTS) [1,2], starch (ST) [3,4], and sodium alginate (ALG) [5,6] are biopolymers commonly used in tissue engineering, including wound healing—due to their biocompatibility, biodegradability, and minimal immune responses upon contact with the human body [7]. However, materials obtained only based on biopolymers as well as their mixtures often exhibit insufficient physicochemical and/or mechanical properties. Therefore, it is necessary to modify such materials through the crosslinking process [8].

Crosslinking is the process that leads to the creation of new bonds in material, which consequently leads to changes in properties, for example, in hydrophilicity or water content [9,10]. It is the most common method of modifying polymeric materials.

Generally, hydrogel networks can be crosslinked through chemical, physical, or enzymatic mechanisms. Chemical crosslinking is widely used because it forms stable covalent bonds, providing strong structural integrity. The main limitation of this approach, however, is the potential cytotoxicity of commonly used crosslinkers (like glutaraldehyde, carbodiimides, or epoxides), which necessitate thorough post-reaction purification to remove unreacted residues [11,12,13]. These safety concerns have driven the development of alternative, biocompatible crosslinking strategies based on naturally derived molecules, including modified polysaccharides.

Modifications of polysaccharides are increasingly used to obtain dialdehyde compounds, typically achieved through periodate oxidation. Dialdehyde starch [14,15,16], dialdehyde chitosan [17,18,19], dialdehyde carboxymethyl cellulose [20,21,22], as well as dialdehyde alginate [23,24] were already applied as promising cross-linking agents for the biopolymeric materials. For example, the modification of collagen with dialdehyde alginate resulted in improvements in thermostability, density, hydrophilicity, and cytocompatibility [23]. Further, fish gelatin films modified with dialdehyde alginate, were characterized by better mechanical strength and higher antioxidant activity than non-modified biopolymer samples [25]. Likewise, dialdehyde alginate-crosslinked casein films exhibited properties distinct from those of pristine samples. Bajpai et al., noticed that a strong correlation exists between the water absorption, moisture permeation and expansion ratio (ER) of the casein crosslinked and non-crosslinked (by dialdehyde alginate) films studied [26]. Additionally, the experiment of J. Li et al. indicated that dialdehyde alginate acted as the green crosslinking agent [27].

Dialdehyde alginate (ADA) has been successfully used to modify various chitosan [28,29,30,31] and carboxymethyl chitosan [32] materials. The previous research compared the crosslinking performance of dialdehyde compounds with that of glutaraldehyde and genipin. It was found that dialdehyde compounds, including ADA, exhibit better cross-linking properties comparable to those of glutaraldehyde. Moreover, they demonstrate high biocompatibility, which cannot be attributed to glutaraldehyde, and they even surpass genipin in this regard [32]. The promising characteristics of ADA, together with its successful application in modifying various biopolymers, motivated us to develop chitosan/starch (CTS/ST) films crosslinked with this agent. CTS/ST systems have already been thoroughly investigated in their native form [33] and in the presence of different crosslinkers (glutaraldehyde [34,35], carboxylic acids [36,37]), as well as plasticizers [38,39,40,41,42].

Considering ADA’s dual advantage of high reactivity and biological tolerance, this study introduces, for the first time, CTS/ST films crosslinked with ADA and evaluates their structural, physicochemical, and biological characteristics to assess their suitability for biomedical applications. The combination of CTS’s amino and hydroxyl groups and ST’s abundant hydroxyl functionalities creates a distinct reactive environment for ADA, enabling the formation of mixed covalent and hydrogen-bonded networks. Such hybrid crosslinking is expected to yield unique properties compared to single-component CST and ST films modified with ADA.

## 2. Results and Discussion

### 2.1. Dialdehyde Alginate Obtaining

**Figure 1:**
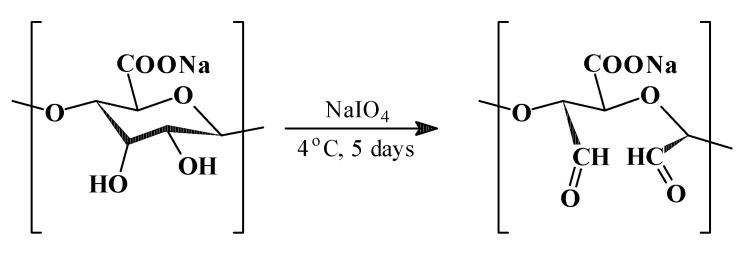
Oxidation of sodium alginate. Formation of aldehyde groups on main chain.

ADA was successfully obtained through the periodate oxidation of sodium alginate (3.0 g) under appropriate reaction conditions (Figure 1). The resulting product exhibited good water solubility. The reaction yield was 2.55 g (ADA1) and 2.64 g (ADA2).

Quantitative analysis of aldehyde group content, performed by hydroxylamine hydrochloride titration, revealed values: 0.843 mol CHO/mol alginate (ADA1) and 0.787 mol CHO/mol alginate (ADA2), depending on the NaIO_4_/monomer molar ratio. Table 1 presents the reagents and reaction parameters of sodium alginate oxidation.

### 2.2. Chitosan/Starch Films Modified with ADA

#### 2.2.1. Fourier Transform Infrared Spectroscopy–Attenuated Total Reflectance

**Figure 2:**
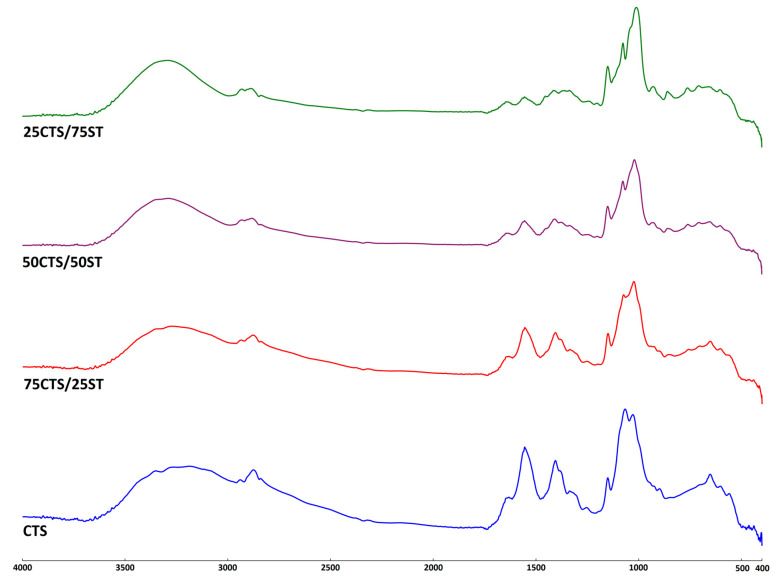
The FTIR-ATR spectra of the materials before crosslinking.

**Figure 3:**
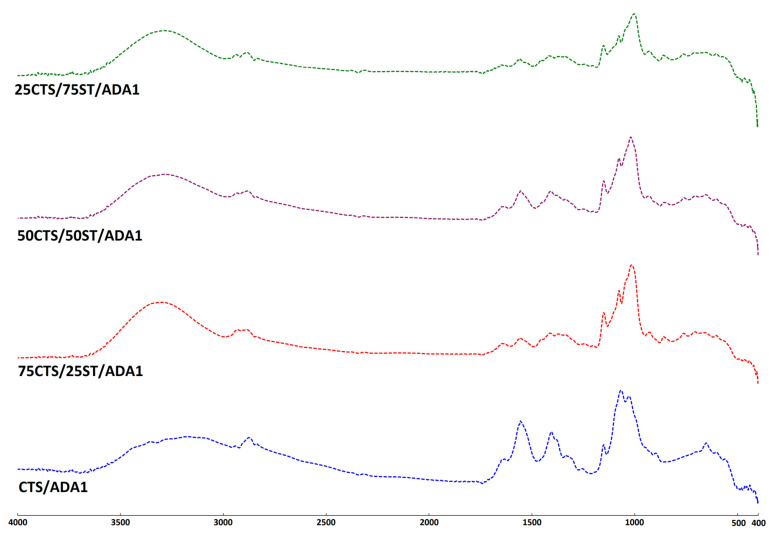
The FTIR-ATR spectra of the examined materials crosslinked with ADA1.

**Figure 4:**
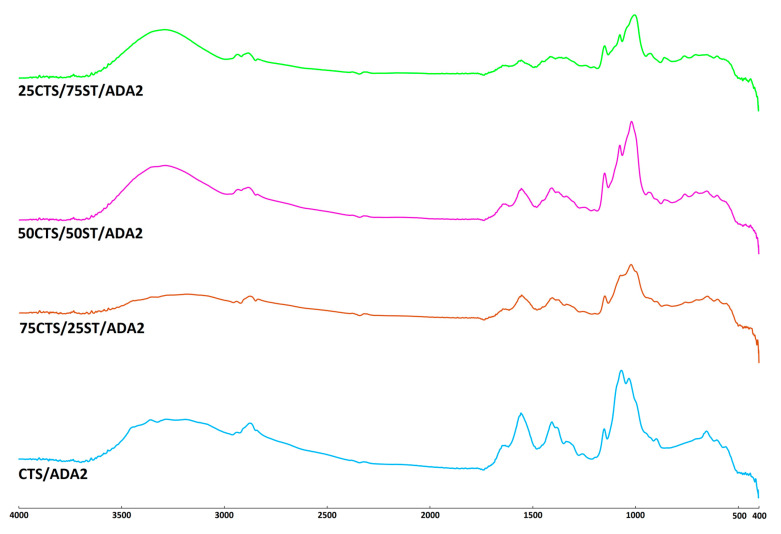
The FTIR-ATR spectra of the examined materials crosslinked with ADA2.

The resulting infrared absorption spectrum provides detailed information about the chemical composition and molecular structure of the material under investigation [43]. In this study, the FTIR-ATR technique was employed to detect the formation of characteristic bonds originating from chitosan and starch, as well as to observe the changes resulting from modification with alginate dialdehyde. The differences between the native and modified materials were evaluated by recording the FTIR spectra of CTS and CTS/ST mixtures, both unmodified and modified with ADA. The spectra of all prepared samples are presented in Figure 2, Figure 3 and Figure 4 (separate spectra for all samples can be found in Supplementary Materials). Additionally, Table 2 shows the specific groups, vibrations and observed changes after ADA addition.

The structures of CTS, ST, and ADA have been previously analyzed and described in the literature [23,30,41,44,45]. As observed, all spectra exhibit a typical broad absorption band between 3600 and 3000 cm^−1^, representing the stretching vibrations of O–H groups, as well as N–H stretching vibrations assigned to the amide A band. The characteristic amide I, II, and III bands were detected at approximately 1630, 1555, and 1406 cm^−1^, respectively. The remaining absorption peaks were attributed to C-H stretching, as well as to C–O–C, C–O, and C–OH vibrations originating from the polysaccharide backbone.

The modification of CTS/ST materials with the addition of ADA1 or ADA2 resulted in slight changes in the position of the peak maxima. The main peak in this region shifted from 3191 cm^−1^ for CTS/ADA1 to 3281 cm^−1^ for 75CTS/25ST/ADA1, 3263 cm^−1^ for 50CTS/50ST/ADA1, and 3276 cm^−1^ for 25CTS/75ST/ADA1. These shifts indicate the formation of hydrogen bonds between the –OH and –NH groups of the material components. A similar observation was made for ADA2.

Slight changes were also observed for the peak with a maximum at 2877 cm^−1^ for CTS/ADA1, which shifted to lower wavenumbers: 2886 cm^−1^ for 75CTS/25ST/ADA1, 2889 cm^−1^ for 50CTS/50ST/ADA1, and 2890 cm^−1^ for 25CTS/75ST/ADA1. Comparable shifts were found for ADA2. No significant shifts were detected in the amide II or amide III regions. However, changes were observed for the peak at 1022 cm^−1^ (corresponding to C–O–C and C–O stretching vibrations of polysaccharides), which shifted to 998 cm^−1^ for 75CTS/25ST/ADA1, 999 cm^−1^ for 50CTS/50ST/ADA1, and 996 cm^−1^ for 25CTS/75ST/ADA1, with similar trends observed for ADA2.

No significant differences were noted between the various CTS/ST ratios; however, the addition of ADA1 or ADA2 suggests that functional groups of the polymers, such as –OH, –NH_2_, and C–O–C, interact with the carboxyl (–COOH) or carbonyl (C=O) groups present in ADA1 and ADA2.

The absorption band observed in the 1630–1640 cm^−1^ region, commonly attributed to Schiff base formation, overlaps with the C=O stretching vibration of the amide I band as well as with absorptions arising from bound water within the biopolymer structure, reflecting the strong moisture-binding capacity of polysaccharides [46,47]. However, comparison of the band shape in this spectral region reveals noticeable changes following ADA addition, including variations in intensity and broadening relative to the unmodified samples. These spectral modifications suggest alterations in the chemical environment of the amino groups and are consistent with the formation of imine (Schiff base) linkages, although the overlap of vibrational modes prevents an unequivocal assignment based solely on this band.

**Figure 5:**
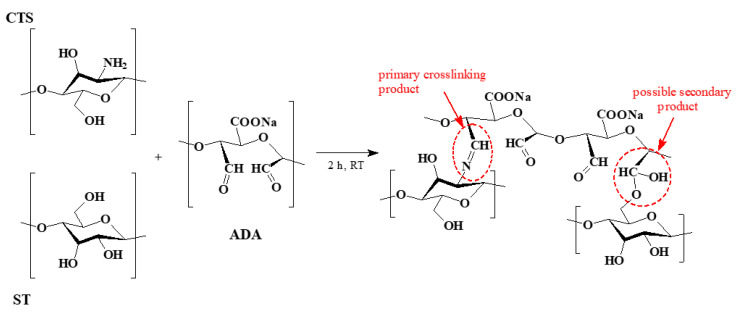
The schematic representation of the reaction products formed during film preparation. Imine-linked crosslinks represent the dominant product, while hemiacetal structures may form to a limited extent under the applied conditions.

Based on the literature, possible cross-linking mechanisms have been proposed, as illustrated in Figure 5 [48,49]. Schiff base formation occurs between the free amino groups of CTS and the aldehyde groups of the dialdehyde compound [45,46], and it is the dominant product of the crosslinking reaction. However, hemiacetal bonds may form between the C=O groups of ADA and the hydroxyl groups of ST [25,50], what was proved between poly(vinyl alcohol) and dialdehyde cellulose [48], but it is expected to be limited under our processing conditions.

#### 2.2.2. Contact Angle

The contact angle, as well as the surface free energy and its dispersive and polar components, are shown in Table 3. Two different liquids, glycerol (G) and diiodomethane (D), were used to obtain the results.

As observed, ADA incorporation does not induce a uniform change in surface wettability across all compositions. In pure CTS films, ADA caused only minor changes in contact angles and surface free energy, likely because the CTS surface is highly homogeneous and dominated by strong intrinsic hydrogen bonding, which limits both surface reorganization and the impact of additional polar groups [51]. In contrast, CTS/ST blends have more heterogeneous and accessible surfaces, allowing ADA-induced crosslinking and polar-group incorporation to produce a more pronounced effect.

In CTS-rich films (75CTS/25ST), where more amino groups are available for Schiff-base formation, ADA addition significantly decreases the G contact angle and increases the D contact angle, resulting in a reduction of total surface energy. Similar observations were reported by Y. Hu et al. for collagen materials modified with dialdehyde alginate [23]. For blends with equal content (50CTS/50ST) or higher ST content (25CTS/75ST), ADA produces negligible effects on the G contact angle; and for the D contact angle, only ADA2 induces changes, increasing it in the 50CTS/50ST and decreasing it in the 25/CTS/75ST. Consequently, the resulting changes in total surface free energy are less pronounced. The differences between ADA1 and ADA2 result primarily from their distinct degree of oxidation but may also arise from the partial depolymerization of alginate during periodate treatment, which cleaves vicinal diols and generates dialdehyde functionalities [23,52,53,54]. This oxidation-induced chain scission increases ADA water solubility and the introduction of additional polar groups [53].

Overall, ADA incorporation decreases the G contact angle only for 75CTS/25ST blend, indicating a slight increase in hydrophilicity, while ADA2 generally increases the D contact angle in CT/ST blends except those with a higher ST content. These effects arise from two opposing mechanisms: the introduction of additional hydroxyl groups [53], which increases polarity and promotes hydrophilicity, and the increase in crosslinking density, which can instead enhance hydrophobic character [23,52,53,54,55]. Collectively, these findings suggest that the polymer ratio, ADA oxidation degree, and competitive interaction among functional groups have a linked influence on the resulting surface wettability.

#### 2.2.3. Moisture Content

Moisture content is an important parameter in the evaluation of wound dressing materials, as it influences their ability to maintain an appropriate wound environment and affects structural stability during application [56]. The results (Table 4) show that pure CTS exhibits the highest moisture content, while the addition of ADA1 and ADA2 reduces it. Incorporation of ST into CTS also decreases the moisture content, with values dropping from 9.07 ± 0.21 mg/100 g for CTS to 1.67 ± 0.40 mg/100 g for 75CTS/25ST, 2.57 ± 0.38 mg/100 g for 50CTS/50ST, and 3.53 ± 0.55 mg/100 g for 25CTS/75ST.

In turn, the effect of ADA on the moisture content in CTS/ST mixtures depended on their composition, and no consistent trend was observed. For the 75CTS/25ST and 50CTS/50ST blends, ADA addition did not cause significant changes, whereas in the 25CTS/75ST sample, it clearly decreased the moisture content compared to the non-modified (from 3.53 ± 0.55 mg/100 g to 2.23 ± 0.06 mg/100 g for ADA1 and 1.97 ± 0.47 mg/100 g for ADA2).

As CTS contains numerous hydrophilic groups (–OH, –NH_2_) capable of binding water, unmodified films retain the highest moisture content [57]. The addition of ADA forms Schiff cross-linking, which reduces the number of free water-binding groups and therefore lowers the moisture content for pure CTS. Interestingly, the lowest moisture content was observed for the 75CTS/25ST blend, whereas a further increase in ST content led to a gradual rise in moisture uptake. This non-monotonic trend likely reflects a composition-dependent reorganization of the polymer network. The CTS/ST interactions may promote a more compact structure with limited free volume available for water due to hydrogen bonding and/or higher ST levels introduce more hydrophilic domains, which facilitates water penetration [58].

Furthermore, the composition also affects the ADA influence on moisture content, which is governed not only by the nominal availability of the reactive -NH_2_ group, but possibly also by the way CTS and ST chains are distributed within the network. In ST-rich films (25CTS/75ST), the ADA-induced rearrangement of the matrix likely enhances the restriction of water uptake, which is consistent with the pronounced decrease in moisture content observed for this composition. Finally, the differences between ADA1 and ADA2 are likely related to their different aldehyde group content [46,59].

#### 2.2.4. Mechanical Testing

**Figure 6:**
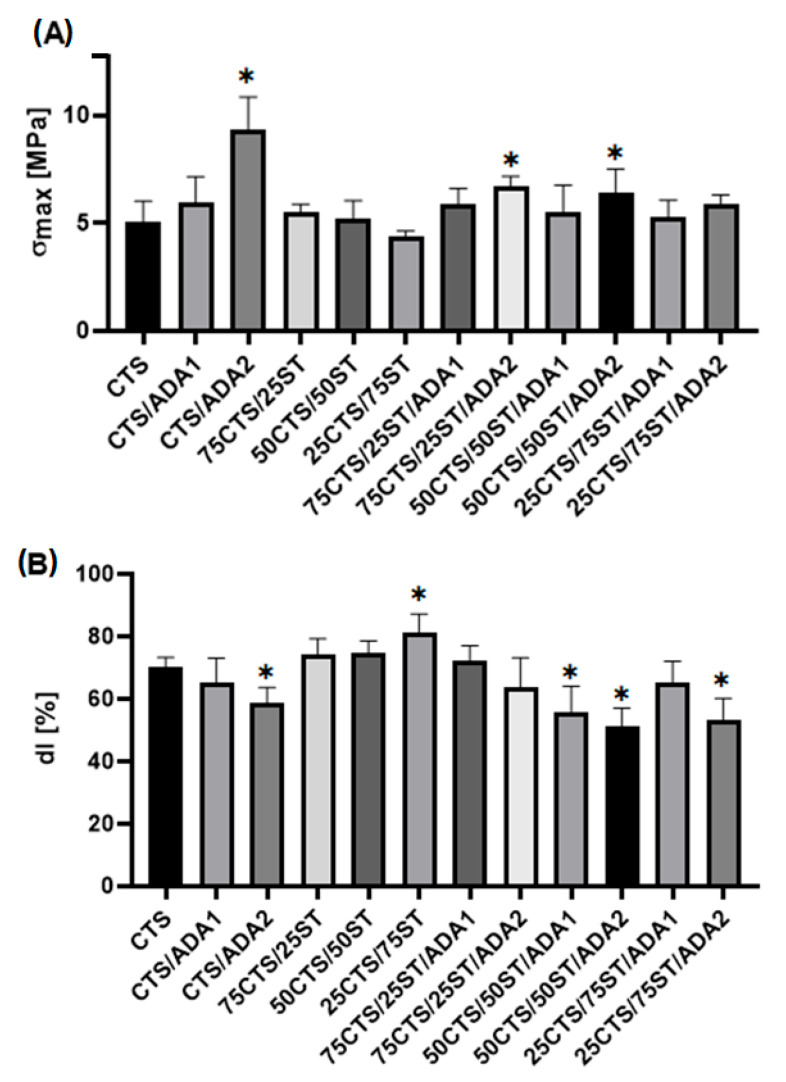
The maximum tensile strength (σ_max_) (**A**) and elongation at break (dl) (**B**) of tested CTS and CTS/ST mixtures modified with ADA; *—significant difference vs. CTS.

The mechanical properties of CTS/ST films were strongly affected by modification with ADA. As shown in Figure 6A, selected formulations exhibited a statistically significant increase in tensile strength (σ_max_) compared with the CTS control, indicating effective reinforcement of the polymer matrix. In particular, CTS/ADA2 showed the highest σ_max_, which can be attributed to the formation of Schiff base linkages between aldehyde groups of ADA and amino groups of chitosan, resulting in enhanced network cohesion. The elongation at break (dl) values (Figure 6B) generally decreased for ADA-modified films, especially for CTS/ADA2, reflecting reduced chain mobility due to increased crosslink density. This inverse relationship between σ_max_ and dl is typical for crosslinked polysaccharide systems [60,61].

#### 2.2.5. Scanning Electron Microscopy (SEM) Imaging

**Figure 7:**
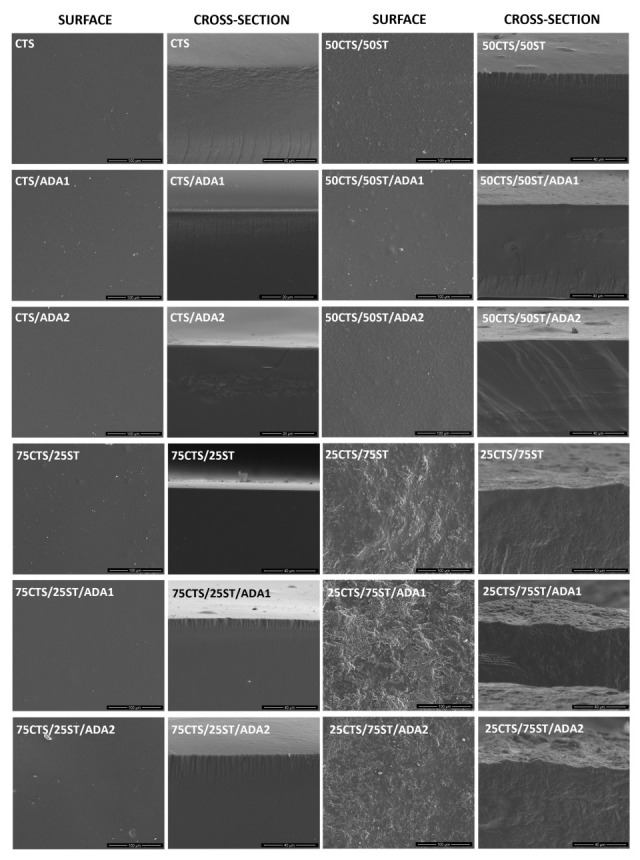
SEM images of surface and cross-sections of examined films.

SEM images of the CTS and CTS/ST films, before and after modification with ADA have been shown in Figure 7. The surface of the reference CTS film is relatively smooth and continuous, without visible cracks or phase-separated domains, indicating effective film formation and good structural integrity of the chitosan matrix [62,63]. Only minor irregularities are observed, which can be attributed to the sample preparation process (breaking after immersion in liquid nitrogen).

The addition of ST to CTS noticeably alters the surface appearance of the films, even in the absence of crosslinking agent (ADA). These changes depend on the film composition. 75CTS/25ST films exhibit only subtle surface modifications compared to pure CTS, whereas increasing the ST content leads to progressively more pronounced surface heterogeneity. The most evident surface alterations are observed for 25CTS/75ST films, where the surface becomes clearly irregular and non-uniform, with a distinct granular-like appearance. These observations indicate a strong influence of ST incorporation on the surface morphology of CTS films, particularly at higher ST content (25CTS/75ST), in non-crosslinked systems.

Generally, the cross-section images reveal no distinct ST granules or large voids, suggesting good dispersion of ST within the CTS matrix and the absence of macroscopic structural discontinuities. However, 25CTS/75ST films display visible irregularities in their internal structure. The cross-sections of these samples differ markedly from the rest of the compositions, showing a less uniform morphology both in non-crosslinked and ADA-crosslinked films. This can indicate that high ST content significantly affects the internal organization of the polymer matrix, regardless of the presence of the crosslinking agent.

Overall, the SEM observations demonstrate that both blending CTS with ST and the introduction of ADA as a crosslinking agent have a pronounced impact on the surface morphology and internal structure of the films. These structural changes arise from interactions between CTS, ST and ADA.

#### 2.2.6. Antioxidant Activity

Wound-healing materials that exhibit antioxidant activity may help regulate the redox balance, reduce inflammation, and promote the progression of the wound into the proliferative phase of healing. Free radical scavenging activity is an effective indicator for assessing the antioxidative properties of thin films with potential biomedical applications. The DPPH radical scavenging assay is considered a standard, simple colorimetric method for evaluating the free-radical scavenging capacity of materials [25,64].

**Figure 8:**
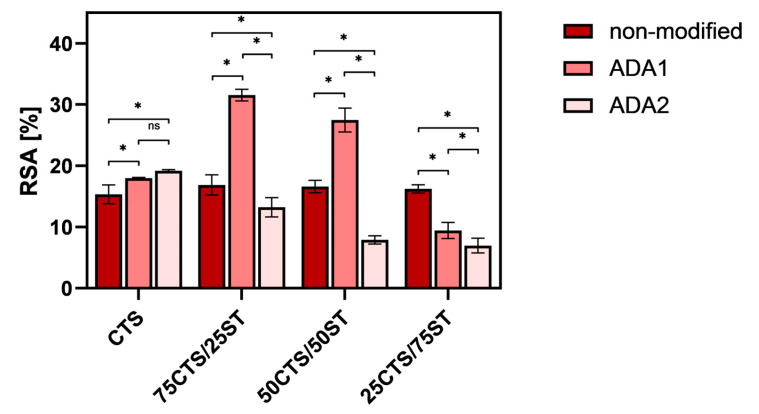
The antioxidant activity of tested CTS and CTS/ST mixtures, before and after modification with ADA; *—significant difference between samples for each group of blends (CTS; 75CTS/25ST; 50CTS/50ST; 25CTS/75ST) (*p* < 0.05); ns—not significant.

The radical scavenging activity of the tested samples is presented in Figure 8. The highest antioxidant activity was observed for two films: 75CTS/25ST/ADA1 and 50CTS/50ST/ADA1. Generally, the addition of ADA affects the antioxidant activity of all sample types. The modification of films with ADA1 typically increases their ability to scavenge free radicals, except for the 25CTS/75ST mixtures, where the RSA is lower for the ADA1-modified sample than for the non-modified sample. The same observations were reported for fish gelatin films modified with ADA [25]. The authors demonstrated that the DPPH and ABTS radical scavenging tests indicated an increase in the antioxidative ability of the fish gelatin/ADA films as ADA content increased and was incorporated. This effect can be hypothesized to result from the formation of bonds between the aldehyde groups and the amino groups during the crosslinking process [25]. It is also known from the scientific literature that chitosan exhibits relatively low radical scavenging activity (RSA) values [65].

However, in the case of ADA2, except for pure CTS, the RSA values are lower than those of the non-modified samples or the samples modified with ADA1. Considering both the composition of the mixtures and the type of dialdehyde alginate (ADA1 or ADA2), the lowest RSA values were obtained for the 25CTS/75ST mixture modified with ADA2.

Although ADA1 contains a higher amount of aldehyde groups than ADA2, the antioxidant activity appears to be governed primarily by the accessibility of chitosan amino groups and the architecture of the crosslinked network, rather than by aldehyde content alone. It should be emphasized that this is a tentative interpretation, as there is currently insufficient experimental evidence to unambiguously identify the underlying cause of these observations. Further studies involving a broader range of oxidation degrees are required to establish a clearer structure–activity relationship.

#### 2.2.7. Blood Hemolysis

Blood compatibility is a crucial parameter in the development of wound dressing materials, as direct contact with blood can affect the integrity of erythrocytes. Hemolysis leads to the release of hemoglobin, and therefore, colorimetric assays are commonly employed to assess the extent of erythrocyte membrane damage. According to the ASTM F756-00 standard [66], materials can be classified as non-hemolytic (hemolytic index between 0 and 2%), slightly hemolytic (2–5%), or hemolytic (>5%) [67].

The hemocompatibility results of the prepared films are presented in Table 4. None of the tested samples exhibited a hemolytic index higher than 2%, which indicates that all films fall within the non-hemolytic category and may be considered safe for potential biomedical applications involving blood contact.

#### 2.2.8. Cytotoxicity Assessment

**Figure 9:**
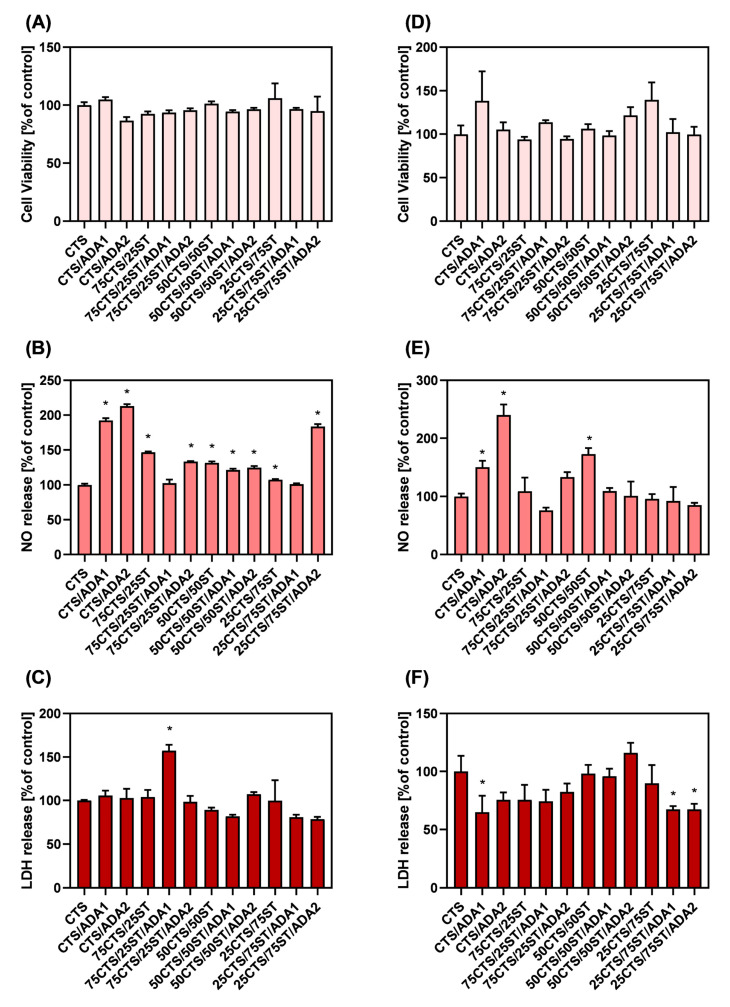
HaCaT keratinocyte viability and release of NO and LDH enzymes. (**A**–**C**)—cells cultured with material extracts; (**D**–**F**)—cells cultured directly on the surface of the films (n = 3; * significantly different from 100% CTS—*p* < 0.05).

One of the fundamental methods for determining a material’s cytocompatibility in vitro is to perform cell viability and cytotoxicity tests [68]. It is also vital to note that NO synthesis plays a major role in the skin. It is crucial for the skin’s reactivity to external stimuli like ultraviolet light, wound healing, and infection [69]. The results of cell viability and release of NO and LDH have been shown in Figure 9.

In the case of direct contact between cells and the surfaces of the tested materials, no significant differences in cellular metabolic activity were observed, and no cytotoxic effect based on LDH release was detected. Cell contact with the material surface induced NO synthesis in certain cases, but no significant relationship between the material composition or its modifications was found.

In the case of cell exposure to material extracts, no alterations in metabolic activity were observed, and only 75CTS/25ST/ADA1 material extract exhibited a cytotoxic effect, as evidenced by an increased presence of LDH in the culture medium. A particularly noteworthy observation is that all of the extracts caused an elevation of NO levels in the culture medium. NO is a molecule of considerable importance for skin physiology. It regulates microcirculation and angiogenesis, facilitates keratinocyte proliferation and epidermal barrier homeostasis, mediates UV-induced melanogenesis and protects against photo-induced apoptosis, exhibits antimicrobial activities, supports wound healing and hair growth, and provides antioxidant protection, counteracting lipid peroxidation and photoaging [70,71,72].

Testing extracts from wound dressing materials on cell viability and metabolic activity in vitro is a fundamental step in assessing their biocompatibility, in accordance with ISO 10993-5 [73], which defines a material as cytotoxic if exposure to its extract reduces cell viability below 70% compared to the control. This approach allows determination of whether the material releases soluble compounds—such as residual monomers, degradation products, or additives—in amounts potentially harmful to cells before being applied in contact with skin or tissue. In practice, these tests involve extracting the material in culture medium (usually 24–72 h at 37 °C), followed by analysis using assays such as the MTS/LDH assay, which quantitatively assesses cell metabolism and survival after exposure [74,75,76].

It is also important to note that materials intended for use as wound dressings should not support metabolic activity that may be related to cell proliferation, since removal of the dressing could otherwise exacerbate the wound by disrupting newly formed tissue [77]. The absence of cytotoxic effects, combined with the stimulation of NO synthesis by the material extracts, makes it a promising candidate for biomedical applications [78,79].

#### 2.2.9. Future Directions Toward Clinical Translation

The obtained findings confirmed that the developed films meet several critical material and performance requirements for wound dressing applications [80], including: (i) hydrophilicity, (ii) controlled moisture balance, (iii) antioxidant activity, (iv) low hemolysis rate, (v) proper cytocompatibility, and (vi) stimulation of NO synthesis. However, further evaluations are required to advance these materials toward clinical translation, particularly with respect to mechanical integrity, oxygen permeability, and long-term biostability. In addition, scalable and reproducible manufacturing must be demonstrated before progressing to animal studies, with particular attention to achieving uniform ADA crosslinking across large-area films, consistent film thickness, and batch-to-batch reproducibility during solvent casting. From a regulatory perspective, wound dressings are classified as medical devices and must comply with ISO10993 biocompatibility requirements [73], including irritation and systemic toxicity testing, as well as validated sterility assurance [81]. Shelf-life stability and the preservation of film properties during storage also require thorough assessment. Collectively, clinical translation is a complex and long-term process, and meeting these performance, manufacturing, and regulatory requirements criteria will be essential for advancing CTS/ST + ADA films toward preclinical and eventual medical application.

## 3. Materials and Methods

### 3.1. Materials

Sodium alginate was obtained from Biomus company (Biomus sp. z o.o., Lublin, Poland), chitosan (low molecular weight; 20–300 cps; 50–190 kDa; 75–85% deacetylated) was purchased from Merck KGaA (Darmstadt, Germany), starch was purchased from Avantor Performance Materials Poland S.A. (Gliwice, Poland). All other starting reagents: sodium periodate, ethylene glycol, sodium chloride, ethanol, acetone, hydrochloric acid, sodium hydroxide, acetic acid, glycerol, were purchased from Aldrich Chemical Co. (Milwaukee, WI, USA).

### 3.2. Oxidation of Sodium Alginate with Sodium Periodate

Several oxidation protocols for sodium alginate reported in the literature [54,82,83,84] were initially screened, and a modified method proposed by Kristiansen et al. [82] was selected. Products with different aldehyde group contents were obtained by applying two different concentrations of periodic acid.

The oxidation reactions were carried out in aqueous solution at 4 °C for 5 days. Firstly, SA (3.0 g) was dissolved in distilled water (270 mL) and stored in a dark bottle. Then, an aqueous solution (30 mL) of sodium periodate (1.62 g, 7.58 mmol for ADA1; and 1.45 g, 6.77 mmol for ADA2) was added to the SA solution, and the reaction mixture was magnetically stirred for 5 days at 4 °C in the dark. After completion, the reaction was quenched by adding ethylene glycol (2.0 mL) and stirring for 1 h at room temperature.

The oxidized alginate was isolated by adding NaCl (1.10 g) and ethanol (500 mL). The obtained polymer was redissolved in water (120 mL) and reprecipitated by the addition of ethanol (250 mL) in the presence of NaCl (0.45 g). Finally, the precipitation was filtered, washed with acetone (20 mL) and dried under vacuum at room temperature.

The aldehyde group content in dialdehyde alginate was determined by colorimetric titration, as described by Zhao et al. [85]. Briefly, this method is based on the reaction of the amino group in hydroxylamine hydrochloride and the aldehyde group, releasing an equimolar amount of hydrochloric acid. The aldehyde content (mol CHO/mol alginate) was quantitatively determined by titrating the released hydrochloric acid with 0.1 M standard NaOH solution.

### 3.3. Processing of Chitosan/Starch Film Modified with ADA

CTS/ST films were prepared using the solvent casting method. A 2% (*w*/*v*) chitosan solution was obtained by dissolving it in a 0.1 mol/L acetic acid solution overnight, while a 2% (*w*/*v*) starch solution was prepared in distilled water under continuous heating and stirring until complete dissolution. Then, both polymer solutions were mixed in a following mass ratios: 75/25, 50/50 and 25/75, followed by stirring for 2 h. Afterward, glycerol was added as a plasticizer at 5% (*w*/*w*) relative to the total solution mass, followed by 1 h of stirring. Subsequently, ADA was incorporated as a crosslinking agent at 5% (*w*/*w*) relative to the total polymer content, and the mixture was stirred for an additional 2 h. The total mixing time was 5 h (2 + 1 + 2).

The final solutions were cast onto square polystyrene plates (10 × 10 cm) and left to dry at room temperature (20–22 °C) under ambient humidity (45–55%) for 72 h to allow solvent evaporation.

### 3.4. Characterization of Starch/Chitosan Films Modified with ADA’

#### 3.4.1. FTIR

The infrared spectra of the films were obtained using a Nicolet iS5 Fourier Transform Infrared spectrometer (Thermo Fisher Scientific, Waltham, MA, USA) equipped with an ID7 ATR accessory and a zinc selenide (ZnSe) crystal. Measurements were performed at room temperature under ambient atmospheric conditions. The spectra were recorded over the wavenumber range of 4000–400 cm^−1^ with a spectral resolution of 4 cm^−1^, and each spectrum represented the average of 32 scans to improve the signal-to-noise ratio.

#### 3.4.2. Contact Angle

The contact angles of the obtained films have been measured at room temperature and ambient humidity using different liquids, including diiodomethane (D) and glycerol (G). The liquid drops were placed onto the thin film surface using a microsyringe, and the resulting images were recorded by a video camera and subsequently digitized. The single-drop profiles were numerically solved and fitted with appropriate mathematical functions using the corresponding instrument software. Each accepted result of the contact angle is the average value of three measurements, with a precision of 0.2°. The surface free energy (SFE), as well as its polar and dispersive components, were calculated using the Owens–Wendt method [86]. The study has been applied by a DSA10 goniometer equipped with a system for drop-shape analysis (Krüss GmbH, Hamburg, Germany).

#### 3.4.3. Moisture Content

The moisture content of the films was measured using a moisture analyzer MA 50.X2.IC.A (Radwag, Radom, Poland). Film specimens (4 cm × 4 cm) were weighed, dried for 10 min at 105 °C, and reweighed. The moisture content was calculated from the weight difference before and after drying and expressed as grams of water per 100 g of dry sample. Measurements were performed in triplicate for each film type.

#### 3.4.4. Mechanical Testing

The mechanical properties of the films were evaluated using a Shimadzu EZ-Test EZ-SX universal testing machine (Shimadzu, Kyoto, Japan). The specimens were secured between clamps and elongated at a crosshead speed of 5 mm·min^−1^. The maximum tensile strength and elongation at break were calculated from the stress–strain curves (n = 10) using Trapezium X Texture software (version 2.0, Kyoto, Japan).

#### 3.4.5. Scanning Electron Microscopy Imaging

The internal morphology of the films was investigated using a Scanning Electron Microscope (SEM; Quanta 3D FEG, FEI, Eindhoven, The Netherlands). The films were fractured after immersion in liquid nitrogen to observe their cross-sections. Prior to imaging, the samples were sputter-coated with a thin layer of gold to ensure electrical conductivity. Micrographs were acquired at a magnification of 1000× (surface) and 2500× (cross-section) for all examined samples.

#### 3.4.6. Antioxidant Activity

The antioxidant activity of the prepared films was assessed using the DPPH (2,2-diphenyl-1-picrylhydrazyl, free radical, 95%; Alfa Aesar, Karlsruhe, Germany) radical scavenging assay [87]. A methanolic solution of DPPH was freshly prepared at a concentration of 250 µM. Film samples (1 × 1 cm) were placed individually into wells of a 12-well plate, followed by the addition of 2 mL of the DPPH solution. The plates were incubated for 1 h in the dark at room temperature to prevent photodegradation of the reagent. A DPPH solution without contact with the films served as a control. After incubation, the absorbance of the solutions was measured at 517 nm using a UV-Vis spectrophotometer (UV-1800, Shimadzu, Muttenz, Switzerland). The radical scavenging activity (RSA, %) was calculated according to the following equation:

(1) $$RSA\%=\frac{{Abs}_{DPPH}-{Abs}_{PB}}{{Abs}_{DPPH}}\times 100\%$$

where Abs_DPPH_ is the absorbance of the control (DPPH solution without film), and Abs_PB_ is the absorbance of the DPPH solution after exposure to the film. All measurements were carried out in triplicate.

#### 3.4.7. Blood Hemolysis

Blood compatibility was assessed using the procedure described by Rahimi et al. [88], with slight modifications. The interaction of the tested materials with blood cells was examined using the direct contact method, in which the release of hemoglobin into plasma serves as an indicator of erythrocyte membrane damage caused by contact with the biomaterial.

Briefly, 0.1 mL of anticoagulated sheep blood was diluted in 5 mL of sterile saline solution (0.9%) containing a 1 cm^2^ specimen of the tested material. The mixtures were incubated at 37 °C for 1 h and subsequently centrifuged at 10,000 rpm for 5 min. Supernatants were transferred into a 96-well plate, and absorbance was recorded at 540 nm using a microplate reader (Multiscan FC; Thermo Fisher Scientific).

Positive and negative controls were prepared by mixing 0.1 mL of sheep blood with distilled water and saline solution, respectively. Each condition was tested in quadruplicate. The percentage of hemolysis was calculated using the following equation:

(2) $$rate\;of\;hemolysis\left[\%\right]=\frac{\left[OD\right]specimen-\left[OD\right]negative}{\left[OD\right]positive-\left[OD\right]negative}\times 100\%$$

#### 3.4.8. Cytotoxicity Assessment

In vitro cytocompatibility evaluation included two experimental setups. The films were sterilized by UV light on both sides. In the experiment, a short UV-C exposure time (15 min, ~254 nm) was applied in a laminar flow hood, which constitutes a standard surface sterilization procedure [89].

In the first system, in order to evaluate the effect of direct contact with cells, HaCaT keratinocyte cells (Thermo Fisher Scientific, Waltham, MA, USA) were cultured in α-MEM supplemented with 10% fetal bovine serum (FBS) and antibiotics (1%) on the surface of the material at a density 2 × 10^4^ cells/cm^2^ for a period of 72 h. The second experiment aimed to investigate the effect of substances released from the material on cell behavior. For this purpose, the HaCaT cells were cultured at the same density on standard culture plastic for 24 h, then they were exposed to extracts obtained as a result of 72 h of incubation of the materials in the culture medium itself. HaCaT keratinocytes were selected for the cytotoxicity study because they represent the primary cell type of the epidermis and are directly involved in the early stages of wound healing expected to result from the material’s action [90,91].

In both systems, the cells were cultured in 24-well plates. To assess the cells viability, the cultures were washed with PBS, and 0.2 mL solution of 10% MTS reagent (CellTiter96Aqueous One Solution Cell Proliferation Assay; Promega, Madison, WI, USA) in phenol-free alpha-MEM was added to individual wells [92,93,94]. The plates were incubated at 37 °C until a noticeable change in color from yellow to brownish occurred. Then, the media were transferred to individual wells in 96-well plates, and the absorbance was recorded at 492 nm using a plate reader Furthermore, the amount of nitric oxide, an indicator of cellular inflammatory response, was detected using a colorimetric Griess reaction in accordance with the protocol. An equal volume of the collected samples (50 μL), Griess A (0.1% N-1-naphthylethylenediamine dihydrochloride), and Griess B (1% sulfanilamide in 5% phosphoric acid; Sigma-Aldrich, St. Louis, MO, USA) were mixed in a 96-well plate. The intensity of the formed color was measured at a wavelength of 540 nm [95]. Finally, a lactate dehydrogenase (LDH) assay (indicator of cell membrane damage) was conducted using a Cytotoxicity Detection Kit (Roche, Mannheim, Germany) as previously described. Briefly, the culture medium was collected, and 50 μL of each sample was placed into a 96-well plate. Then, an equal amount of reagent mixture prepared according to the manufacturer’s instructions was mixed with the samples. After incubation at 37 °C, the intensity of the red color formed in the colorimetric assay was measured at a wavelength of 490 nm [95].

### 3.5. Statistical Analysis

Statistical analysis of the data was completed using commercial software (GraphPad Prism 8.0.1.244, GraphPad Software, San Diego, CA, USA). The results were presented as the mean ± standard deviation (SD) and were statistically analyzed using a one-way analysis of variance (one-way ANOVA). Multiple comparisons between the means were performed with the statistical significance set at *p* ≤ 0.05.

For biological testing, the experiments were performed in either triplicate or quadruplicate. The statistically significant differences were assessed using one-way ANOVA and a post hoc Tukey test, with *p* < 0.05 considered significant.

## 4. Conclusions

Films based on chitosan and starch prepared in different mass ratios (75/25, 50/50, and 25/75) were successfully obtained and modified with dialdehyde alginate containing two different contents of aldehyde groups (0.843 and 0.787 mol CHO/mol alginate). FTIR analysis confirmed the presence of characteristic functional groups of the constituent biopolymers, as well as the modification of films by ADA crosslinking, and affected bands shifts. The ADA modification influenced the water content of the films, generally reducing moisture uptake due to the additional network formation. Films crosslinked with ADA1 exhibited improved antioxidant activity in the DPPH assay, which may be attributed to the presence of reactive aldehyde-derived structures. Furthermore, the contact angle results showed tunable wettability depending on the CTS/ST ratio and aldehyde content, which may be advantageous for tailoring surface interactions in biomedical applications. Hemolysis testing revealed good hemocompatibility across all films, while in vitro cytocompatibility studies demonstrated that the films supported cell viability.

Overall, the obtained results suggest that the proposed chitosan/starch films cross-linked with dialdehyde alginate represent favorable physicochemical properties, antioxidant potential, and biocompatibility. These findings highlight their suitability for further development toward wound dressing and related biomedical applications.

## Figures and Tables

**Table 1:** The reagents and reaction parameters of sodium alginate oxidation.

Reaction Parameters	ADA1	ADA2
Sodium periodate used for oxidation [g]	1.62	1.45
Sodium periodate used for oxidation [mmol]	7.58	6.77
Reaction yield [g]	2.55	2.64
Aldehyde groups content [mol CHO/mol alginate]	0.843	0.787

**Table 2:** FTIR-ATR peak assignments of CTS and CTS/ST blends, before and after modification with ADA.

Wavenumber [cm^−1^]	Vibrational Mode	Functional Group	Observed Changes After ADA Addition
3600–3000	Stretching vibration	O–H, N–H (amide A)	Shift to higher wavenumbers; band broadening
~3280–3190	Stretching vibration	O–H/N–H	Composition-dependent peak shift
~2877–2890	Stretching vibration	C–H	Slight shift to higher wavenumbers
~1630–1640	Stretching vibration	C=O (amide I), C=N (imine), bound water	Band shape broadening; intensity changes
~1555	Bending vibration	N–H (amide II)	No significant shift
~1406	Stretching/bending	C–N/N–H (amide III)	No significant shift
~1022–996	Stretching vibration	C–O–C, C–O	Shift to lower wavenumbers
~890–950	Ring vibration	C–O–C	Minor intensity changes

**Table 3:** The results of contact angles, surface free energy (IFT (s)), and its dispersive (IFT (s,D)) and polar (IFT (s,P)) components of the tested films (n = 5).

Sample	Contact Angle [°]		IFT (s) [mJ/m^2^]	IFT (s,D) [mJ/m^2^]	IFT (s,P) [mJ/m^2^]
	Glycerol	Diiodomethane			
CTS	53.75 ± 1.74	89.97 ± 1.08	42.99 ± 0.48	4.78 ± 0.10	38.22 ± 0.38
CTS/ADA1	54.37 ± 1.89 ^ns^	86.22 ± 2.42 ^ns^	41.29 ± 0.80	6.16 ± 0.25	35.13 ± 0.55
CTS/ADA2	53.90 ± 1.18 ^ns^	83.04 ± 2.19 ^ns^	40.94 ± 0.68	7.37 ± 0.25	33.57 ± 0.43
75CTS/25ST	62.50 ± 2.60	72.26 ± 2.16	33.65 ± 0.79	13.61 ± 0.34	20.04 ± 0.45
75CTS/25ST/ADA1	51.40 ± 2.86 *	76.63 ± 2.19 *	41.86 ± 0.91	9.90 ± 0.30	31.96 ± 0.61
75CTS/25ST/ADA2	51.38 ± 1.48 *	93.65 ± 1.79 *	46.68 ± 0.60	3.46 ± 0.14	43.22 ± 0.46
50CTS/50ST	59.87 ± 2.02	85.34 ± 1.54	36.32 ± 0.59	7.07 ± 0.18	29.25 ± 0.41
50CTS/50ST/ADA1	53.82 ± 1.73 ^ns^	86.53 ± 2.79 ^ns^	41.87 ± 0.88	5.99 ± 0.29	35.87 ± 0.59
50CTS/50ST/ADA2	57.92 ± 2.17 ^ns^	93.37 ± 2.30 *	40.22 ± 0.77	4.04 ± 0.20	36.18 ± 0.57
25CTS/75ST	55.12 ± 2.31	90.14 ± 2.86	41.77 ± 0.94	4.84 ± 0.27	36.94 ± 0.67
25CTS/75ST/ADA1	58.76 ± 2.69 ^ns^	88.00 ± 2.47 ^ns^	37.85 ± 0.87	5.93 ± 0.26	31.93 ± 0.62
25CTS/75ST/ADA2	55.34 ± 1.87 ^ns^	82.90 ± 2.71 *	39.69 ± 0.87	7.58 ± 0.31	32.11 ± 0.56

*—significant difference versus control sample for each group of blends (CTS; 75CTS/25ST; 50CTS/50ST; 25CTS/75ST) (*p* < 0.05); ns—not significant.

**Table 4:** The moisture content and hemolysis rate of CTS and CTS/ST films, before and after modification with ADA; *—significantly different from the control sample within each blend group (CTS; 75CTS/25ST; 50CTS/50ST; 25CTS/75ST) (*p* < 0.05); ns—not significant; ^#^—significantly different from CTS (*p* < 0.05); n = 3.

Sample	Moisture Content [mg/100 g of Dry Sample]	Hemolysis Rate [%]
CTS	9.07 ± 0.21	0.08 ± 0.04
CTS/ADA1	6.87 ± 0.50 *	0.68 ± 0.05 ^#^
CTS/ADA2	5.43 ± 0.74 *	0.59 ± 0.05 ^#^
75CTS/25ST	1.67 ± 0.40	1.15 ± 0.06 ^#^
75CTS/25ST/ADA1	2.23 ± 0.15 ^ns^	0.83 ± 0.22 ^#^
75CTS/25ST/ADA2	2.13 ± 0.15 ^ns^	0.88 ± 0.06 ^#^
50CTS/50ST	2.57 ± 0.38	0.85 ± 0.02 ^#^
50CTS/50ST/ADA1	3.13 ± 0.15 ^ns^	0.64 ± 0.18 ^#^
50CTS/50ST/ADA2	2.23 ± 0.06 ^ns^	0.71 ± 0.27 ^#^
25CTS/75ST	3.53 ± 0.55	1.40 ± 0.22 ^#^
25CTS/75ST/ADA1	2.23 ± 0.06 *	0.00
25CTS/75ST/ADA2	1.97 ± 0.47 *	0.00

## Data Availability

The authors declare that all data supporting the findings of this study are available within the paper; source data for the figures in this study are available from the authors upon request.

## Supplementary Materials

The following supporting information can be downloaded at https://www.mdpi.com/article/10.3390/ijms27031174/s1.

## References

[1] Khan M.U.A., Hakkarainen M., Bin Abdullah M.F., Tayebi L., Gul H., Hasan A. (2025). Recent Perspective of Chitosan in Wound Healing Approaches—A Review. Mater. Today Commun..

[2] Shah J., Patel D., Rananavare D., Hudson D., Tran M., Schloss R., Langrana N., Berthiaume F., Kumar S. (2025). Recent Advancements in Chitosan-Based Biomaterials for Wound Healing. J. Funct. Biomater..

[3] Palanisamy C.P., Cui B., Zhang H., Gunasekaran V.P., Ariyo A.L., Jayaraman S., Rajagopal P., Long Q. (2022). A Critical Review on Starch-Based Electrospun Nanofibrous Scaffolds for Wound Healing Application. Int. J. Biol. Macromol..

[4] Waghmare V.S., Wadke P.R., Dyawanapelly S., Deshpande A., Jain R., Dandekar P. (2018). Starch Based Nano-fibrous Scaffolds for Wound Healing Applications. Bioact. Mater..

[5] Aderibigbe B., Buyana B. (2018). Alginate in Wound Dressings. Pharmaceutics.

[6] Feketshane Z., Adeyemi S.A., Ubanako P., Ndinteh D.T., Ray S.S., Choonara Y.E., Aderibigbe B.A. (2023). Dis-solvable Sodium Alginate-Based Antibacterial Wound Dressing Patches: Design, Characterization, and in Vitro Biological Studies. Int. J. Biol. Macromol..

[7] Gobi R., Ravichandiran P., Babu R.S., Yoo D.J. (2021). Biopolymer and Synthetic Polymer-Based Nanocomposites in Wound Dressing Applications: A Review. Polymers.

[8] Grabska-Zielińska S., Sionkowska A. (2021). How to Improve Physico-chemical Properties of Silk Fibroin Materials for Biomedical Applications?—Blending and Cross-linking of Silk Fibroin—A Review. Materials.

[9] Moghadas B., Solouk A., Sadeghi D. (2020). Development of Chitosan Membrane Using Non-Toxic Crosslinkers for Potential Wound Dressing Applications. Polym. Bull..

[10] Panchal R., Mateti T., Likhith K., Rodrigues F.C., Thakur G. (2022). Genipin Cross-Linked Chitosan–PVA Compo-site Films: An Investigation on the Impact of Cross-Linking on Accelerating Wound Healing. React. Funct. Polym..

[11] Thakur G., Rodrigues F.C., Singh K. (2018). Crosslinking Biopolymers for Advanced Drug Delivery and Tissue Engineering Applications. Adv. Exp. Med. Biol..

[12] Oryan A., Kamali A., Moshiri A., Baharvand H., Daemi H. (2018). Chemical Crosslinking of Biopolymeric Scaffolds: Current Knowledge and Future Directions of Crosslinked Engineered Bone Scaffolds. Int. J. Biol. Macromol..

[13] Grabska-Zielińska S. (2024). Cross-Linking Agents in Three-Component Materials Dedicated to Biomedical Appli-cations: A Review. Polymers.

[14] Sionkowska A., Michalska-Sionkowska M., Walczak M. (2020). Preparation and Characterization of Colla-gen/Hyaluronic Acid/Chitosan Film Crosslinked with Dialdehyde Starch. Int. J. Biol. Macromol..

[15] Skopinska-Wisniewska J., Wegrzynowska-Drzymalska K., Bajek A., Maj M., Sionkowska A. (2016). Is Dialdehyde Starch a Valuable Cross-Linking Agent for Collagen/Elastin Based Materials?. J. Mater. Sci. Mater. Med..

[16] Grabska-Zielińska S., Sionkowska A., Reczyńska K., Pamuła E. (2020). Physico-Chemical Characterization and Biological Tests of Collagen/Silk Fibroin/Chitosan Scaffolds Cross-Linked by Dialdehyde Starch. Polymers.

[17] Grabska-Zielińska S., Sionkowska A., Olewnik-Kruszkowska E., Reczyńska K., Pamuła E. (2021). Is Dialdehyde Chitosan a Good Substance to Modify Physicochemical Properties of Biopolymeric Materials?. Int. J. Mol. Sci..

[18] Bam P., Bhatta A., Krishnamoorthy G. (2019). Design of Biostable Scaffold Based on Collagen Crosslinked by Dial-dehyde Chitosan with Presence of Gallic Acid. Int. J. Biol. Macromol..

[19] Hemsri S., Junwattarunggu N., Rueangsawang W., Aramsrithum S. (2023). Fabrication and Properties of Gela-tin/Dialdehyde Chitosan Films. IOP Conf. Ser. Mater. Sci. Eng..

[20] Yi Y., Zhang Y., Mansel B., Wang Y.N., Prabakar S., Shi B. (2022). Effect of Dialdehyde Carboxymethyl Cellulose Cross-Linking on the Porous Structure of the Collagen Matrix. Biomacromolecules.

[21] Jiang X., Yang Z., Peng Y., Han B., Li Z., Li X., Liu W. (2016). Preparation, Characterization and Feasibility Study of Dialdehyde Carboxymethyl Cellulose as a Novel Crosslinking Reagent. Carbohydr. Polym..

[22] Mu C., Guo J., Li X., Lin W., Li D. (2012). Preparation and Properties of Dialdehyde Carboxymethyl Cellulose Crosslinked Gelatin Edible Films. Food Hydrocoll..

[23] Hu Y., Liu L., Gu Z., Dan W., Dan N., Yu X. (2014). Modification of Collagen with a Natural Derived Cross-Linker, Alginate Dialdehyde. Carbohydr. Polym..

[24] Chen L., Deng X., Tian L., Xie J., Xiang Y., Liang X., Jiang L., Jiang L. (2024). Preparation and Properties of Chi-tosan/Dialdehyde Sodium Alginate/Dopamine Magnetic Drug-Delivery Hydrogels. Colloids Surf. A Physicochem. Eng. Asp..

[25] Park J., Nam J., Yun H., Jin H.J., Kwak H.W. (2021). Aquatic Polymer-Based Edible Films of Fish Gelatin Cross-linked with Alginate Dialdehyde Having Enhanced Physicochemical Properties. Carbohydr. Polym..

[26] Bajpai S.K., Bajpai M., Shah F.F. (2016). Alginate Dialdehyde (AD)-Crosslinked Casein Films: Synthesis, Charac-terization and Water Absorption Behavior. Des. Monomers Polym..

[27] Li J., Zheng Y., Wang P., Zhang H. (2024). The Alginate Dialdehyde Crosslinking on Curcumin-Loaded Zein Nan-ofibers for Controllable Release. Food Res. Int..

[28] Huang J., Zajforoushan Moghaddam S., Maroni P., Thormann E. (2020). Swelling Behavior, Interaction, and Elec-trostatic Properties of Chitosan/Alginate Dialdehyde Multilayer Films with Different Outermost Layer. Langmuir.

[29] Huang J., Moghaddam S.Z., Thormann E. (2020). Chitosan/Alginate Dialdehyde Multilayer Films with Modulated PH-Responsiveness and Swelling. Macromol. Chem. Phys..

[30] Aston R., Wimalaratne M., Brock A., Lawrie G., Grøndahl L. (2015). Interactions between Chitosan and Alginate Dialdehyde Biopolymers and Their Layer-by-Layer Assemblies. Biomacromolecules.

[31] Dong J., Yu D., Zhang L., Wang G., Zhang P., You Y., Xu Y., Xia W. (2024). Chitosan/Alginate Dialdehyde Trilayer Films with Cinnamaldehyde Nanoemulsions for Grass Carp Preservation. Food Hydrocoll..

[32] Wang W., Huang W.C., Zheng J., Xue C., Mao X. (2023). Preparation and Comparison of Dialdehyde Derivatives of Polysaccharides as Cross-Linking Agents. Int. J. Biol. Macromol..

[33] Sun K.Q., Li F.Y., Li J.Y., Li J.F., Zhang C.W., Chen S., Sun X., Cui J.F. (2019). Optimisation of Compatibility for Improving Elongation at Break of Chitosan/Starch Films. RSC Adv..

[34] Li H., Gao X., Wang Y., Zhang X., Tong Z. (2013). Comparison of Chitosan/Starch Composite Film Properties before and after Cross-Linking. Int. J. Biol. Macromol..

[35] Li F., Chen Y., Li L., Bai X., Li S. (2012). Starch-Chitosan Blend Films Prepared by Glutaraldehyde Cross-Linking. Adv. Mater. Res..

[36] Wu H., Lei Y., Lu J., Zhu R., Xiao D., Jiao C., Xia R., Zhang Z., Shen G., Liu Y. (2019). Effect of Citric Acid Induced Crosslinking on the Structure and Properties of Potato Starch/Chitosan Composite Films. Food Hydrocoll..

[37] Bisla V., Yoshitake H. (2024). Control of Mechanical and Hydrophobic Properties of Silylated Chitosan-Starch Films by Cross-Linking Using Carboxylic Acids. Carbohydr. Polym. Technol. Appl..

[38] Tuhin M.O., Rahman N., Haque M.E., Khan R.A., Dafader N.C., Islam R., Nurnabi M., Tonny W. (2012). Modifi-cation of Mechanical and Thermal Property of Chitosan–Starch Blend Films. Radiat. Phys. Chem..

[39] Bangyekan C., Aht-Ong D., Srikulkit K. (2006). Preparation and Properties Evaluation of Chitosan-Coated Cassava Starch Films. Carbohydr. Polym..

[40] Bourtoom T., Chinnan M.S. (2008). Preparation and Properties of Rice Starch-Chitosan Blend Biodegradable Film. LWT-Food Sci. Technol..

[41] Ren L., Yan X., Zhou J., Tong J., Su X. (2017). Influence of Chitosan Concentration on Mechanical and Barrier Properties of Corn Starch/Chitosan Films. Int. J. Biol. Macromol..

[42] Mathew S., Brahmakumar M., Abraham T.E. (2006). Microstructural Imaging and Characterization of the Mechan-ical, Chemical, Thermal, and Swelling Properties of Starch–Chitosan Blend Films. Biopolymers.

[43] Olewnik-Kruszkowska E., Gierszewska M., Wrona M., Nerin C., Grabska-Zielińska S. (2022). Polylactide-Based Films with the Addition of Poly(Ethylene Glycol) and Extract of Propolis—Physico-Chemical and Storage Properties. Foods.

[44] Karua C.S., Sahoo A. (2020). Synthesis and Characterization of Starch/Chitosan Composites. Mater. Today Proc..

[45] Jejurikar A., Seow X.T., Lawrie G., Martin D., Jayakrishnan A., Grøndahl L. (2012). Degradable Alginate Hydrogels Crosslinked by the Macromolecular Crosslinker Alginate Dialdehyde. J. Mater. Chem..

[46] Sarker B., Papageorgiou D.G., Silva R., Zehnder T., Gul-E-Noor F., Bertmer M., Kaschta J., Chrissafis K., Detsch R., Boccaccini A.R. (2014). Fabrication of Alginate–Gelatin Crosslinked Hydrogel Microcapsules and Evalu-ation of the Microstructure and Physico-Chemical Properties. J. Mater. Chem. B.

[47] Gao C., Wang S., Liu B., Yao S., Dai Y., Zhou L., Qin C., Fatehi P. (2021). Sustainable Chitosan-Dialdehyde Cel-lulose Nanocrystal Film. Materials.

[48] Muchová M., Münster L., Capáková Z., Mikulcová V., Kuřitka I., Vícha J. (2020). Design of Dialdehyde Cellulose Crosslinked Poly(Vinyl Alcohol) Hydrogels for Transdermal Drug Delivery and Wound Dressings. Mater. Sci. Eng. C.

[49] Elhag M., Abdelwahab H.E., Mostafa M.A., Yacout G.A., Nasr A.Z., Dambruoso P., El Sadek M.M. (2021). One Pot Synthesis of New Cross-Linked Chitosan-Schiff’ Base: Characterization, and Anti-Proliferative Activities. Int. J. Biol. Macromol..

[50] Dalei G., Das S., Pradhan M. (2022). Dialdehyde Cellulose as a Niche Material for Versatile Applications: An Overview. Cellulose.

[51] Tangpasuthadol V., Pongchaisirikul N., Hoven V.P. (2003). Surface Modification of Chitosan Films.: Effects of Hy-drophobicity on Protein Adsorption. Carbohydr. Res..

[52] Palhares L.C.G.F., London J.A., Kozlowski A.M., Esposito E., Chavante S.F., Ni M., Yates E.A. (2021). Chemical Modification of Glycosaminoglycan Polysaccharides. Molecules.

[53] Balakrishnan B., Mohanty M., Umashankar P.R., Jayakrishnan A. (2005). Evaluation of an in Situ Forming Hydrogel Wound Dressing Based on Oxidized Alginate and Gelatin. Biomaterials.

[54] Balakrishnan B., Jayakrishnan A. (2005). Self-Cross-Linking Biopolymers as Injectable in Situ Forming Biodegradable Scaffolds. Biomaterials.

[55] Liu X., Dan N., Dan W., Gong J. (2016). Feasibility Study of the Natural Derived Chitosan Dialdehyde for Chemical Modification of Collagen. Int. J. Biol. Macromol..

[56] Kozłowska J., Skopińska-Wiśniewska J., Kaczmarek-Szczepańska B., Grabska-Zielińska S., Maku-rat-Kasprolewicz B., Michno A., Ronowska A., Wekwejt M. (2023). Gelatin and Gelatin/Starch-Based Films Modi-fied with Sorbitol for Wound Healing. J. Mech. Behav. Biomed. Mater..

[57] Tsai H.-S., Wang Y.-Z. (2008). Properties of Hydrophilic Chitosan Network Membranes by Introducing Binary Crosslink Agents. Polym. Bull..

[58] Wan Yusof W.R., Sabar S., Zailani M.A. (2024). Starch-Chitosan Blends: A Comprehensive Review on the Prepara-tion, Physicochemical Properties and Applications. Biopolymers.

[59] Tang R., Du Y., Fan L. (2003). Dialdehyde Starch-Crosslinked Chitosan Films and Their Antimicrobial Effects. J. Polym. Sci. B Polym. Phys..

[60] Lewandowska K. (2026). Influence of Cross-Linking Agents on the Structure and Stability of Chitosan and Car-boxymethyl Chitosan Thin Films. Molecules.

[61] Khadsai S., Janmanee R., Sam-Ang P., Nuanchawee Y., Rakitikul W., Mankhong W., Likittrakulwong W., Ninjiaranai P. (2024). Influence of Crosslinking Concentration on the Properties of Biodegradable Modified Cassava Starch-Based Films for Packaging Applications. Polymers.

[62] Arzate-Vázquez I., Chanona-Pérez J.J., Calderón-Domínguez G., Terres-Rojas E., Garibay-Febles V., Mar-tínez-Rivas A., Gutiérrez-López G.F. (2012). Microstructural Characterization of Chitosan and Alginate Films by Microscopy Techniques and Texture Image Analysis. Carbohydr. Polym..

[63] Lewandowska K., Sionkowska A., Kaczmarek B., Furtos G. (2014). Characterization of Chitosan Composites with Various Clays. Int. J. Biol. Macromol..

[64] Comino-Sanz I.M., López-Franco M.D., Castro B., Pancorbo-Hidalgo P.L. (2021). The Role of Antioxidants on Wound Healing: A Review of the Current Evidence. J. Clin. Med..

[65] Kaczmarek-Szczepańska B., Zasada L., Grabska-Zielińska S. (2022). The Physicochemical, Antioxidant, and Color Properties of Thin Films Based on Chitosan Modified by Different Phenolic Acids. Coatings.

[66] (2000). Standard Practice for Assessment of Hemolytic Properties of Materials.

[67] Kaczmarek-Szczepańska B., Michalska-Sionkowska M., Binkowski P., Lukaszewicz J.P., Kamedulski P. (2023). 3D-Structured and Blood-Contact-Safe Graphene Materials. Int. J. Mol. Sci..

[68] Rogero S.O., Malmonge S.M., Lugão A.B., Ikeda T.I., Miyamaru L., Cruz Á.S. (2003). Biocompatibility Study of Polymeric Biomaterials. Artif. Organs.

[69] Cals-Grierson M.M., Ormerod A.D. (2004). Nitric Oxide Function in the Skin. Nitric Oxide.

[70] Adler B.L., Friedman A.J. (2015). Nitric Oxide Therapy for Dermatologic Disease. Future Sci. OA.

[71] Man M., Wakefield J.S., Mauro T.M., Elias P.M. (2022). Role of Nitric Oxide in Regulating Epidermal Permeability Barrier Function. Exp. Dermatol..

[72] Zaborova V., Budanova E., Kryuchkova K., Rybakov V., Shestakov D., Isaikin A., Romanov D., Chur-yukanov M., Vakhnina N., Zakharov V. (2025). Nitric Oxide: A Gas Transmitter in Healthy and Diseased Skin. Med. Gas Res..

[73] (2009). Biological Evaluation of Medical Devices—Part 5: Tests for In Vitro Cytotoxicity.

[74] Montufar E.B. (2025). Bone Response to Biodegradable Metals and In Vitro Evaluation of the Cytocompatibility. JOM.

[75] Paula A.B., Laranjo M., Coelho A.S., Abrantes A.M., Gonçalves A.C., Sarmento-Ribeiro A.B., Ferreira M.M., Botelho M.F., Marto C.M., Carrilho E. (2021). Accessing the Cytotoxicity and Cell Response to Biomaterials. J. Vis. Exp..

[76] Jhamb S.K., Goyal A., Pandey A., Bhowmik A. (2025). A Review of Cytotoxicity Testing Methods and in Vitro Study of Biodegradable Mg-1%Sn-2%HA Composite by Elution Method. J. Mater. Sci. Mater. Med..

[77] Wiegand C., Abel M., Hipler U.-C., Elsner P. (2019). Effect of Non-Adhering Dressings on Promotion of Fibroblast Proliferation and Wound Healing in Vitro. Sci. Rep..

[78] Ahmed R., Augustine R., Chaudhry M., Akhtar U.A., Zahid A.A., Tariq M., Falahati M., Ahmad I.S., Hasan A. (2022). Nitric Oxide-Releasing Biomaterials for Promoting Wound Healing in Impaired Diabetic Wounds: State of the Art and Recent Trends. Biomed. Pharmacother..

[79] Alexander-Sinclair E.I., Lapina E.S., Edomenko N.V., Kostyakov D.V., Zinoviev E.V., Blinova M.I., Mikhailova N.A. (2025). Biocompatibility Issues of Wound Dressings. Bioengineering.

[80] Lagoa T., Queiroga M.C., Martins L. (2024). An Overview of Wound Dressing Materials. Pharmaceuticals.

[81] Thanusha A.V., Koul V. (2021). Biocompatibility Evaluation for the Developed Hydrogel Wound Dressing—ISO-10993-11 Standards—In Vitro and in Vivo Study. Biomed. Phys. Eng. Express.

[82] Kristiansen K.A., Tomren H.B., Christensen B.E. (2011). Periodate Oxidized Alginates: Depolymerization Kinetics. Carbohydr. Polym..

[83] Gomez C.G., Rinaudo M., Villar M.A. (2007). Oxidation of Sodium Alginate and Characterization of the Oxidized Derivatives. Carbohydr. Polym..

[84] Salem D.M.S.A., Sallam M.A.E., Youssef T.N.M.A. (2019). Synthesis of Compounds Having Antimicrobial Activity from Alginate. Bioorg. Chem..

[85] Zhao H., Heindel N.D. (1991). Determination of Degree of Substitution of Formyl Groups in Polyaldehyde Dextran by the Hydroxylamine Hydrochloride Method. Pharm. Res. Off. J. Am.-Sociation Pharm. Sci..

[86] Owens D.K., Wendt R.C. (1969). Estimation of the Surface Free Energy of Polymers. J. Appl. Polym. Sci..

[87] Grabska-Zielińska S., Gierszewska M., Olewnik-Kruszkowska E., Bouaziz M.M.A. (2021). Polylactide Films with the Addition of Olive Leaf Extract—Physico-Chemical Characterization. Materials.

[88] Rahimi M., Ahmadi R., Samadi Kafil H., Shafiei-Irannejad V. (2019). A Novel Bioactive Quaternized Chitosan and Its Silver-Containing Nanocomposites as a Potent Antimicrobial Wound Dressing: Structural and Biological Properties. Mater. Sci. Eng. C.

[89] Suh D., Sherlock S.H., Dukes K.C., Perencevich E.N., Marra A.R. (2025). Impact of UV-C on Material Degradation: A Scoping Literature Review. Antimicrob. Steward. Healthc. Epidemiol. ASHE.

[90] Witte M.B., Barbul A. (2002). Role of Nitric Oxide in Wound Repair. Am. J. Surg..

[91] Boukamp P., Petrussevska R.T., Breitkreutz D., Hornung J., Markham A., Fusenig N.E. (1988). Normal Keratini-zation in a Spontaneously Immortalized Aneuploid Human Keratinocyte Cell Line. J. Cell Biol..

[92] Kaczmarek B., Nadolna K., Owczarek A., Mazur O., Sionkowska A., Łukowicz K., Vishnu J., Manivasagam G., Osyczka A.M. (2020). Properties of Scaffolds Based on Chitosan and Collagen with Bioglass 45S5. IET Nanobiotechnol..

[93] Kaczmarek-Szczepańska B., D’Amora U., Zasada L., Michalska-Sionkowska M., Miłek O., Łukowicz K., Osyczka A.M. (2025). Enhancing Thin Film Properties of Chitosan–Collagen Biocomposites Through Potassium Sil-icate and Tannic Acid Integration. Polymers.

[94] Łukowicz K., Zagrajczuk B., Wieczorek J., Millan-Ciesielska K., Polkowska I., Cholewa-Kowalska K., Osyczka A.M. (2022). Molecular Indicators of Biomaterials Osteoinductivity—Cell Migration, BMP Production and Signalling Turns a Key. Stem Cell Rev. Rep..

[95] Kaczmarek-Szczepańska B., Glajc P., Chmielniak D., Gwizdalska K., Swiontek Brzezinska M., Dembińska K., Shinde A.H., Gierszewska M., Łukowicz K., Basta-Kaim A. (2025). Development and Characterization of Biocompatible Chitosan-Aloe Vera Films Functionalized with Gluconolactone and Sorbitol for Advanced Wound Healing Applications. ACS Appl. Mater. Interfaces.

