# Supplementary material for: Dialdehyde Alginate as a Crosslinker for Chitosan/Starch Films: Toward Biocompatible and Antioxidant Wound Dressing Materials
Source: Int J Mol Sci. 2026 Jan 23;27(3):1174. doi: 10.3390/ijms27031174 (PMC12897587; doi:10.3390/ijms27031174)
Supplement: Supplementary file 1 [file ijms-27-01174-s001.zip › ijms-4015297-supplementary.pdf]

International Journal of *Molecular Sciences*

MDPI

Supplementary Materials

Dialdehyde Alginate as a Crosslinker for Chitosan/Starch Films: Toward Biocompatible and Antioxidant Wound Dressing Materials

Sylwia Grabska–Zielińska [1,*], Marek Pietrzak [1], Lidia Zasada [2], Krzysztof Łukowicz [3,4], Agnieszka Basta–Kaim [3], Marta Michalska–Sionkowska [5], Marcin Wekwejt [6], Beata Kaczmarek–Szczepańska [2,*]

1 Faculty of Chemical Technology and Engineering, Bydgoszcz University of Science and Technology, Seminaryjna 3, 85-326 Bydgoszcz, Poland; sylwia.grabska-zielinska@pbs.edu.pl (S.G.-Z.); marek.pietrzak@pbs.edu.pl (M.P)
2 Laboratory for Functional Polymeric Materials, Faculty of Chemistry, Nicolaus Copernicus University in Torun, Gagarin 7, 87-100 Toruń, Poland; beata.kaczmarek@umk.pl (B.K.-S.); 503555@doktorant.umk.pl (L. Z.)
3 Laboratory of Immunoendocrinology, Department of Experimental Neuroendocrinology, Maj Institute of Pharmacology, Polish Academy of Sciences, 12 Smętna St., Kraków, 31-343, Poland; lukowicz@if-pan.krakow.pl
4 Department of Medical Physics, Cyclotron Centre Bronowice, Institute of Nuclear Physics, Polish Academy of Sciences, Radzikowskiego 152, Kraków, 31-342, Poland
5 Department of Environmental Microbiology and Biotechnology, Faculty of Biological and Veterinary Sciences, Nicolaus Copernicus University in Toruń, Lwowska 1, 87-100 Toruń, Poland; mms@umk.pl
6 Department of Biomaterials Technology, Faculty of Mechanical Engineering and Ship Technology, Gdańsk University of Technology, Gdańsk, Poland; marcin.wekwejt@pg.edu.pl
* Correspondence: sylwia.grabska-zielinska@pbs.edu.pl (S.G.-Z.) and beata.kaczmarek@umk.pl (B.K.-S.)

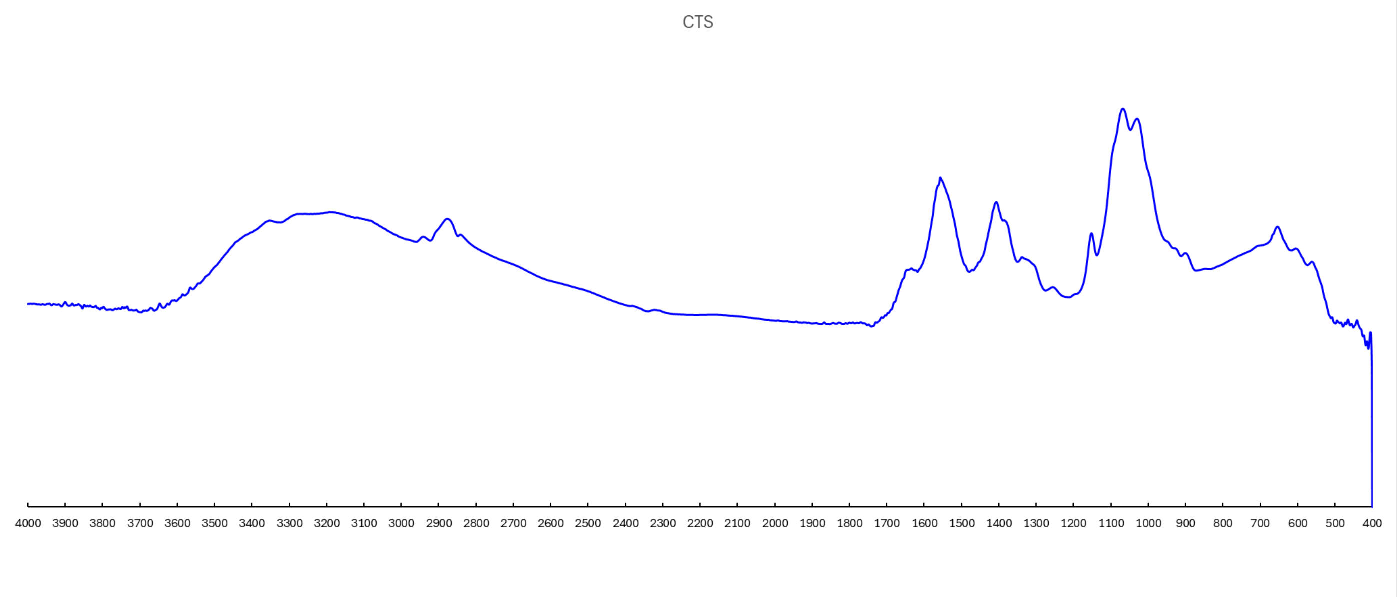

Figure S1. The FTIR spectra of CTS.

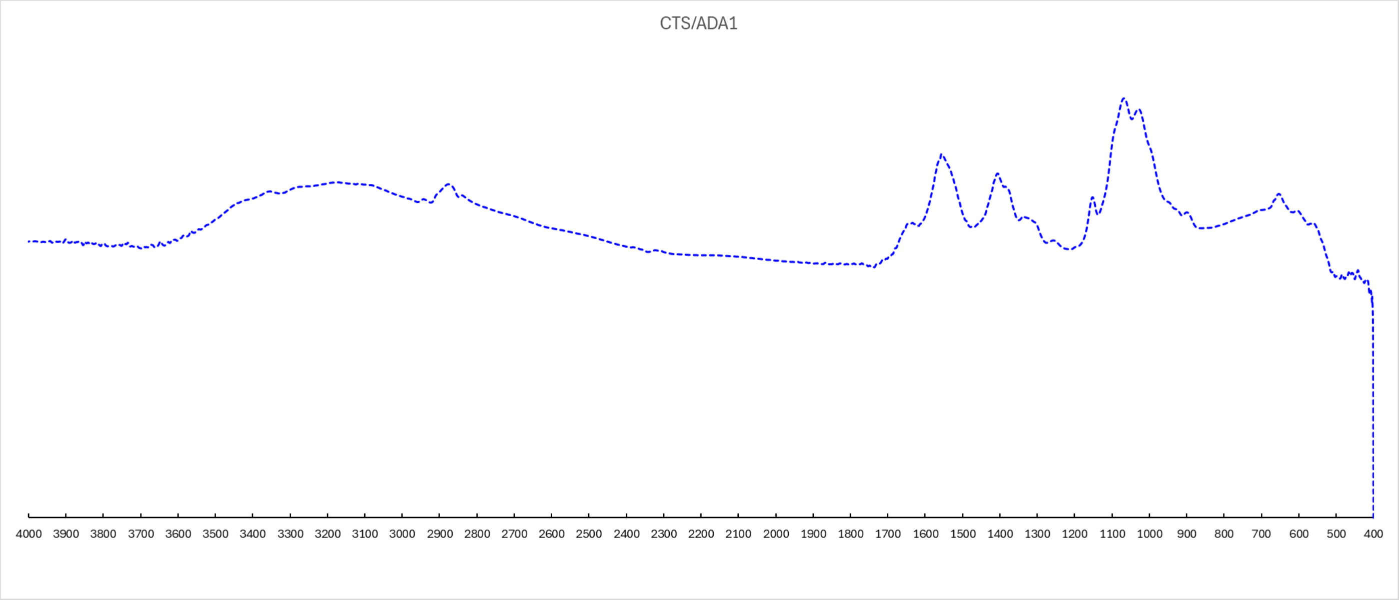

Figure S2. The FTIR spectra of CTS/ADA1.

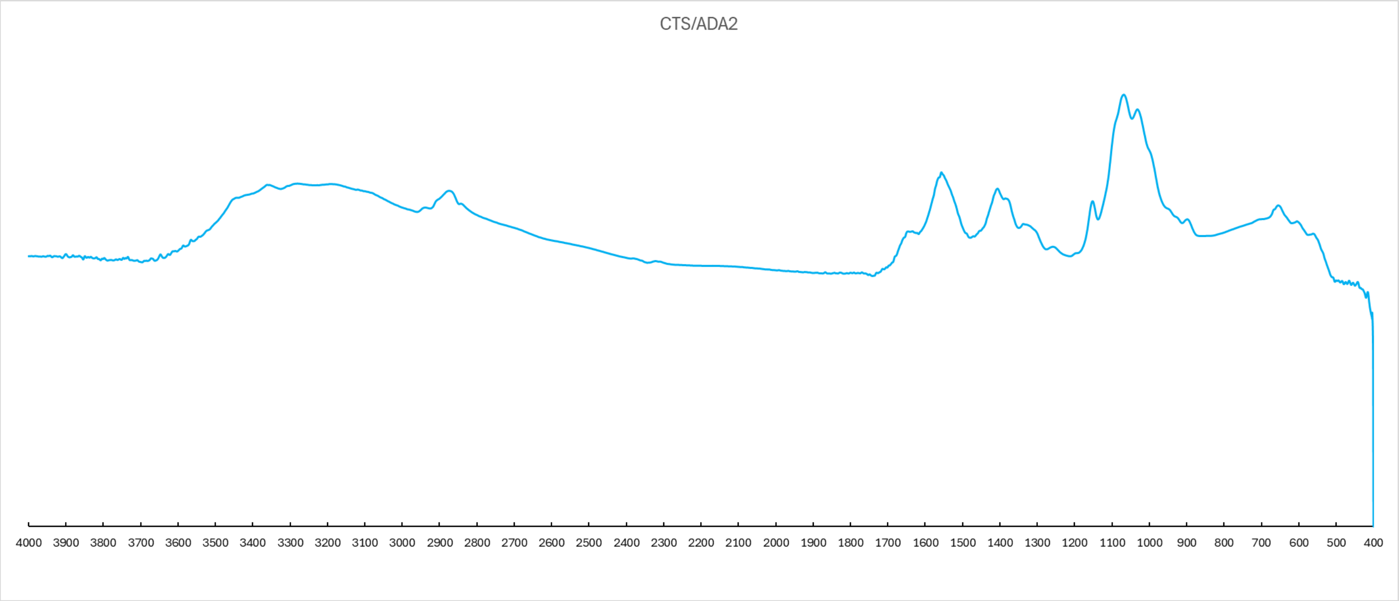

Figure S3. The FTIR spectra of CTS/ADA2.

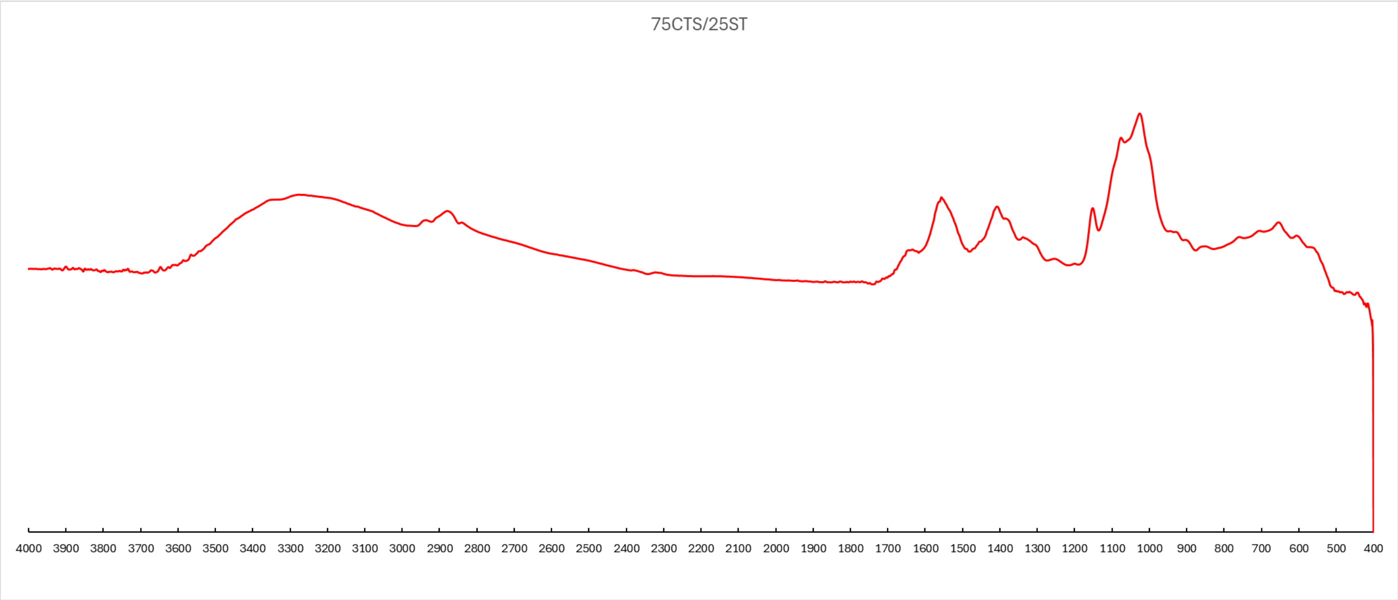

Figure S4. The FTIR spectra of 75CTS/25ST.

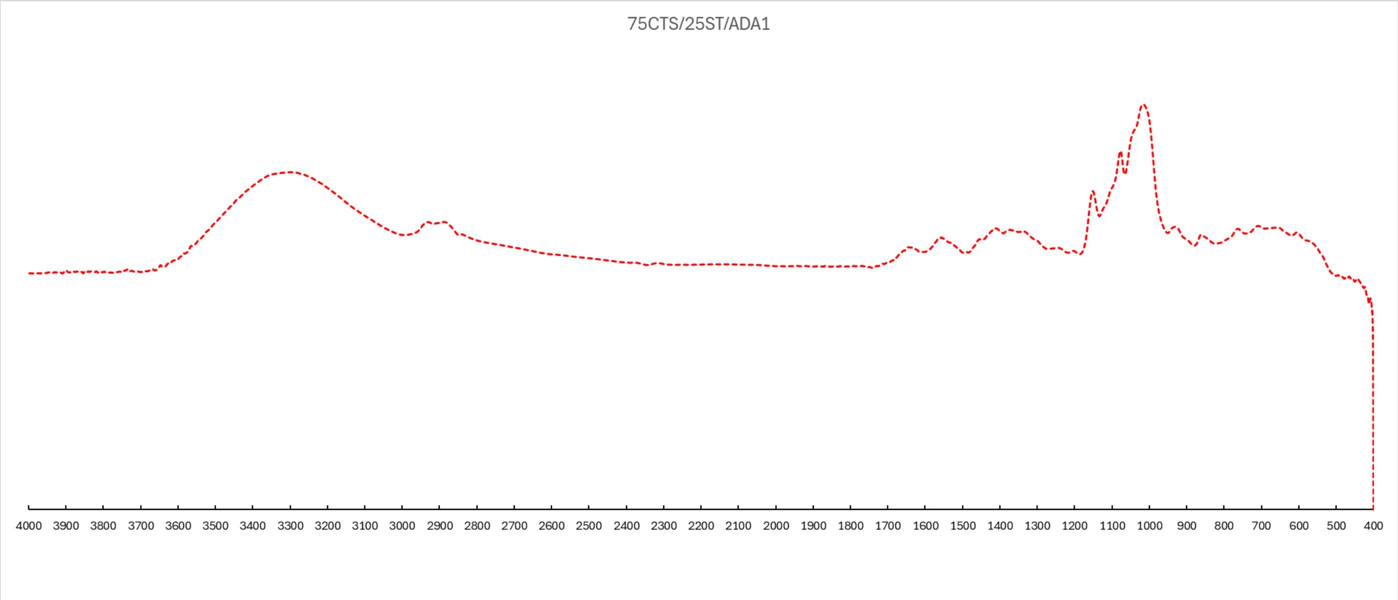

Figure S5. The FTIR spectra of 75CTS/25ST/ADA1.

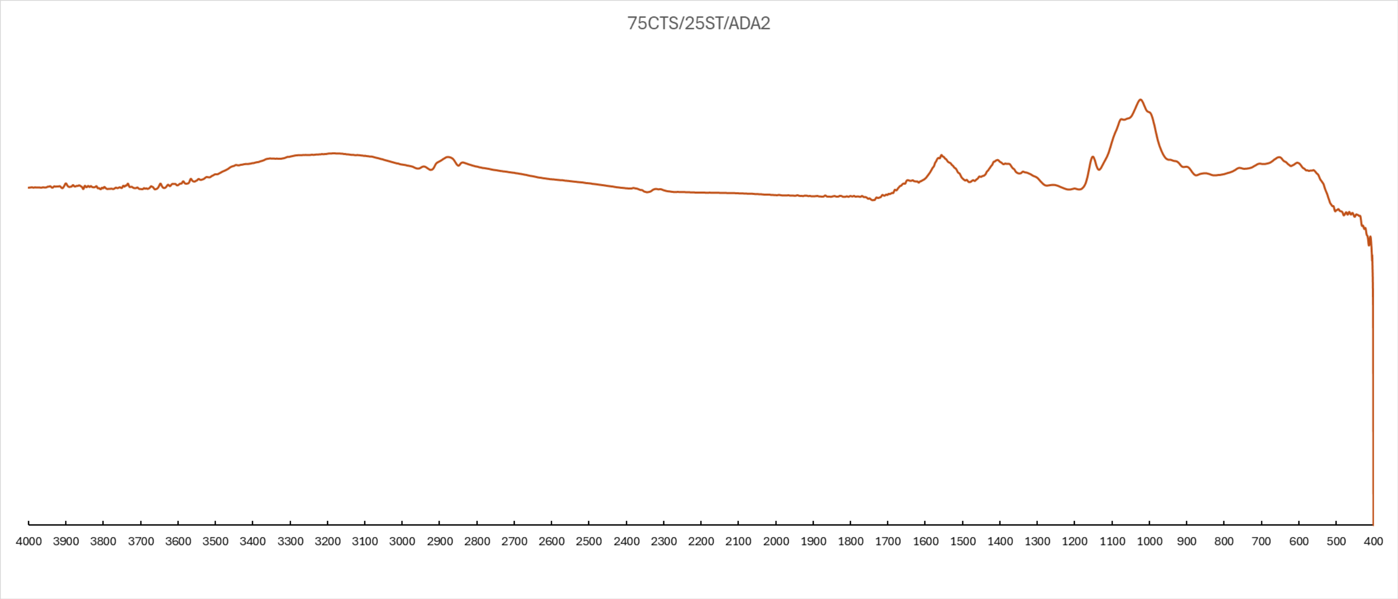

Figure S6. The FTIR spectra of 75CTS/25ST/ADA2.

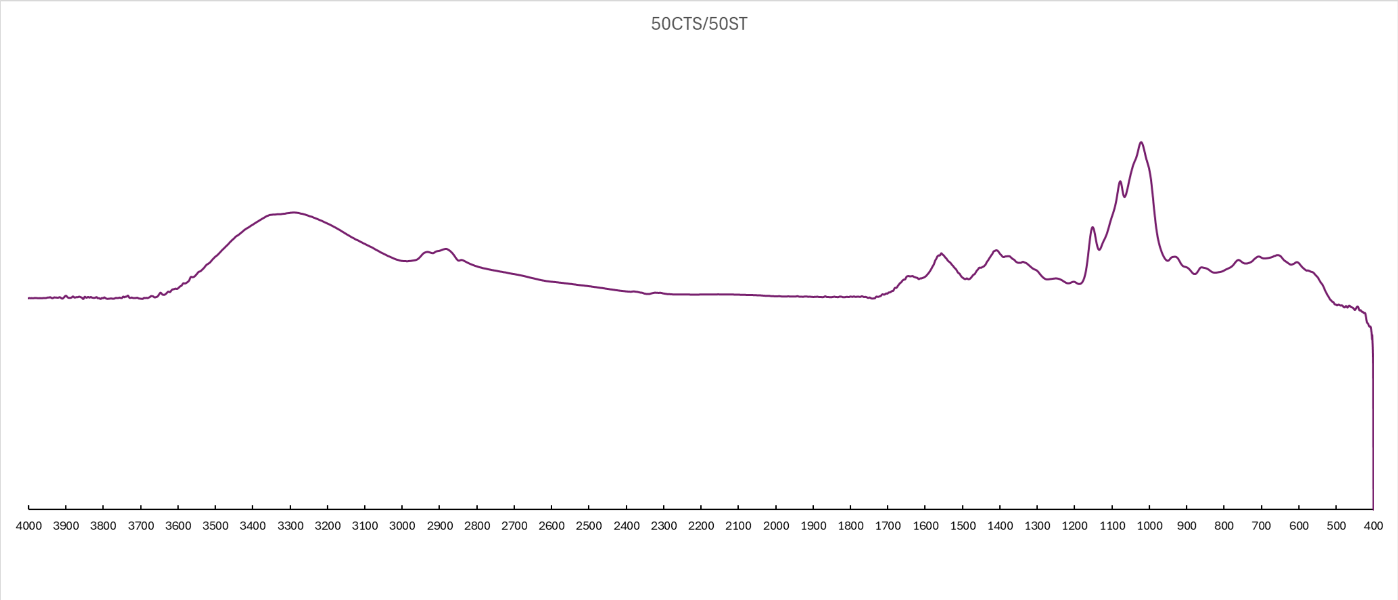

Figure S7. The FTIR spectra of 50CTS/50ST.

https://doi.org/10.3390/ijms27031174

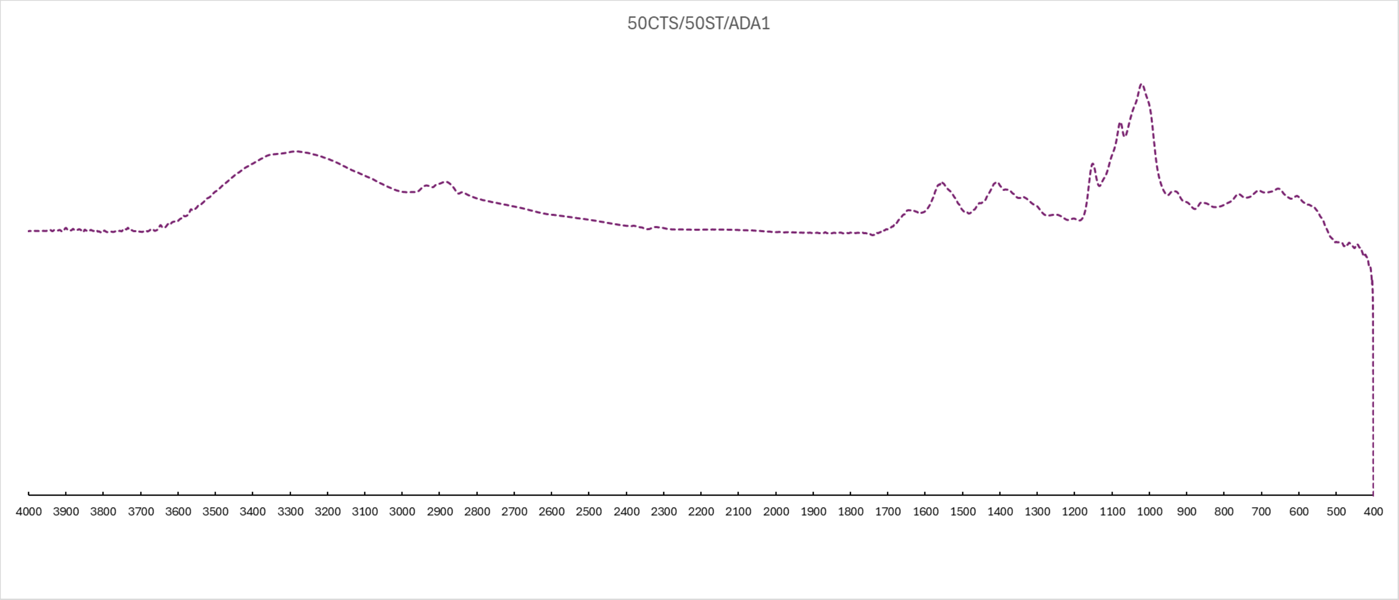

Figure S8. The FTIR spectra of 50CTS/50ST/ADA1.

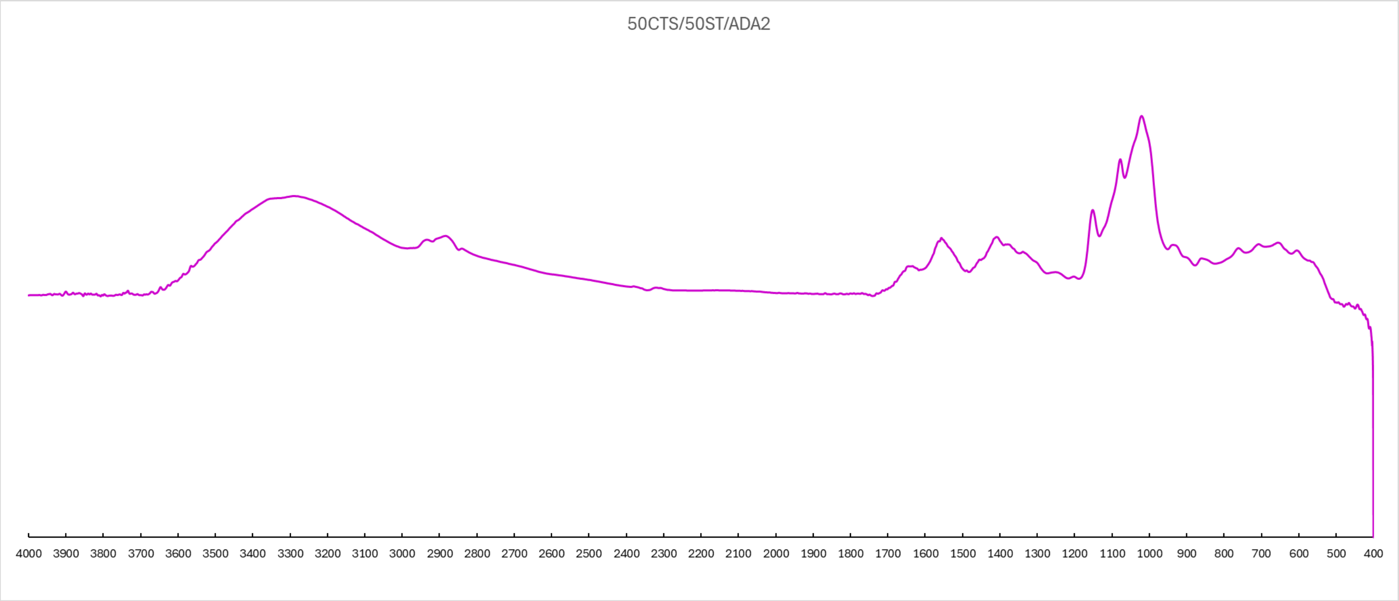

Figure S9. The FTIR spectra of 50CTS/50ST/ADA2.

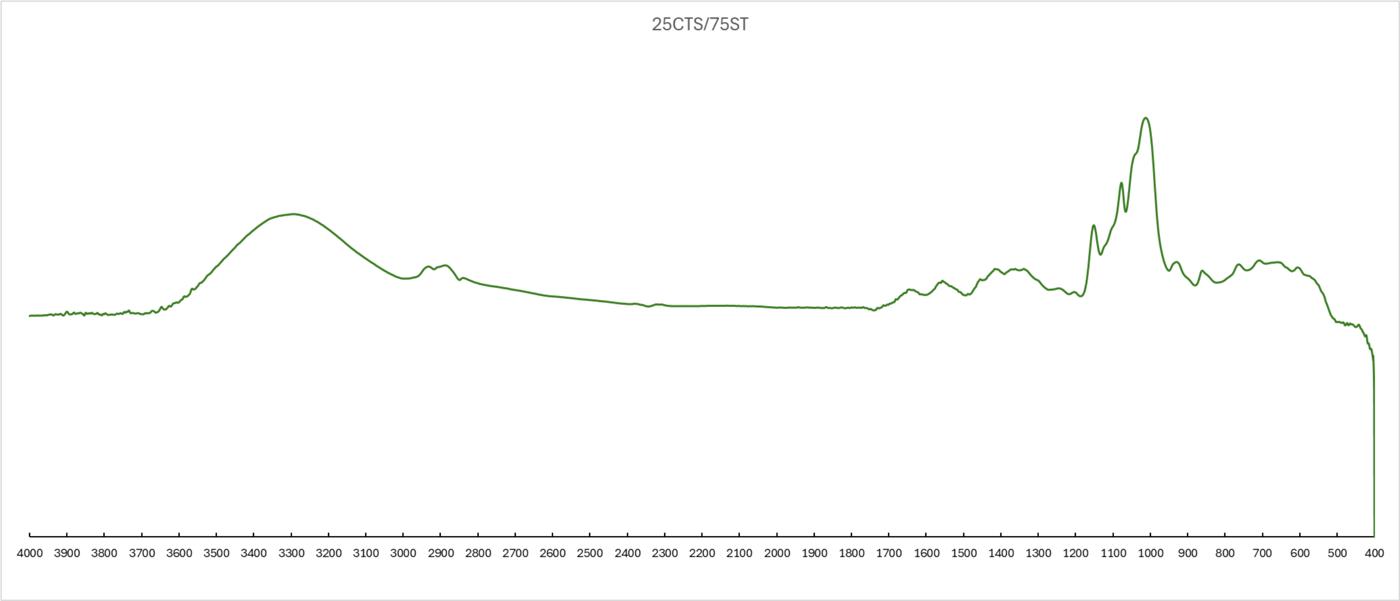

Figure S10. The FTIR spectra of 25CTS/75ST.

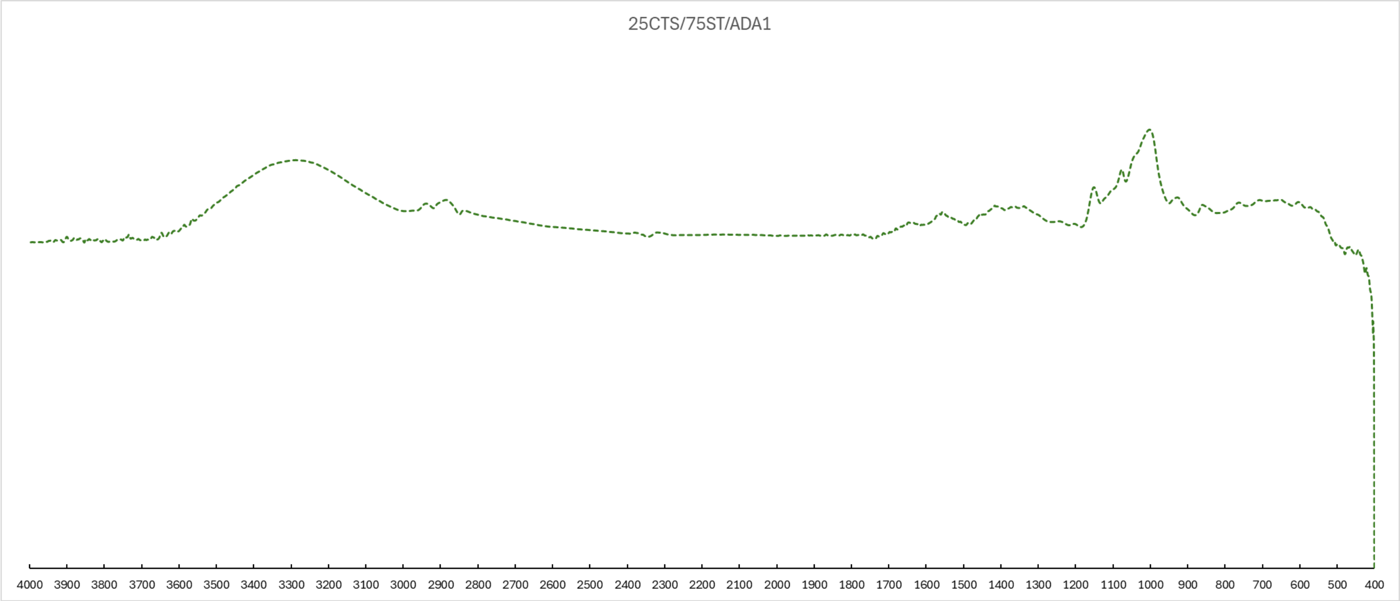

Figure S11. The FTIR spectra of 25CTS/75ST/ADA1.

https://doi.org/10.3390/ijms27031174

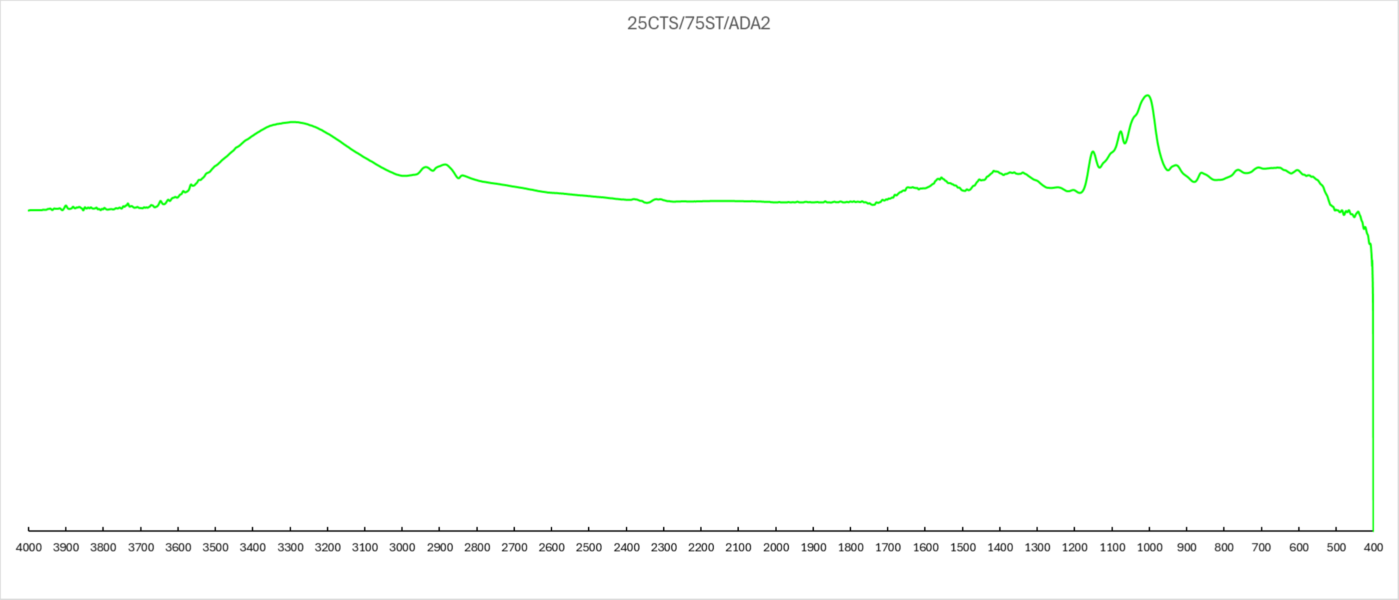

Figure S12. The FTIR spectra of 25CTS/75ST/ADA2.
